# An evolutionarily conserved motif is required for Plasmodesmata-located protein 5 to regulate cell-to-cell movement

**DOI:** 10.1038/s42003-020-1007-0

**Published:** 2020-06-05

**Authors:** Xu Wang, Gabriel Robles Luna, Cecilia Noemi Arighi, Jung-Youn Lee

**Affiliations:** 1 Department of Plant and Soil Sciences, University of Delaware, Newark, DE 19711 USA; 2 Department of Computer and Information Sciences, University of Delaware, Newark, DE 19711 USA; 3 Delaware Biotechnology Institute, University of Delaware, Newark, DE 19711 USA; 4 Present Address: Department of Plant Physiology and Biochemistry, University of Hohenheim, Stuttgart, Germany

**Keywords:** Protein trafficking in plants, Plant molecular biology

## Abstract

Numerous cell surface receptors and receptor-like proteins (RLPs) undergo activation or deactivation via a transmembrane domain (TMD). A subset of plant RLPs distinctively localizes to the plasma membrane-lined pores called plasmodesmata. Those RLPs include the *Arabidopsis thaliana* Plasmodesmata-located protein (PDLP) 5, which is well known for its vital function regulating plasmodesmal gating and molecular movement between cells. In this study, we report that the TMD, although not a determining factor for the plasmodesmal targeting, serves essential roles for the PDLP5 function. In addition to its role for membrane anchoring, the TMD mediates PDLP5 self-interaction and carries an evolutionarily conserved motif that is essential for PDLP5 to regulate cell-to-cell movement. Computational modeling-based analyses suggest that PDLP TMDs have high propensities to dimerize. We discuss how a specific mode(s) of TMD dimerization might serve as a common mechanism for PDLP5 and other PDLP members to regulate cell-to-cell movement.

## Abstract (web summary)

Wang, Robles-Luna et al demonstrate that in Arabidopsis, the transmembrane domain (TMD) of plasmodesmata-located protein 5 (PDLP5) is required for PDPL5 dimerisation and membrane anchoring. This study suggests the importance of the TMD in the role for PDPL5 in regulating pasmodesmal opening.

## Introduction

Plasmodesmata are membrane-lined cytoplasmic nanochannels through which various molecules move between cells. Soluble molecules such as small nutrients, hormones, and ions, move by simple diffusion, whereas some specialized macromolecules through active transport. Among the latter type, some were shown to act as mobile signals essential for specific physiological responses or developmental programming^1,2^. Moreover, during defense against microbial pathogens, plants must find a balance between closing plasmodesmata to limit pathogen spread and access to nutrients, while still allowing defense signals to move locally and systemically. However, while a large amount of information is available about mobile molecules, relatively little is known about the regulatory molecules and mechanisms vital for plasmodesmal control.

We have previously shown that the *Arabidopsis thaliana* Plasmodesmata-located protein (PDLP) 5 localizes to the central region of plasmodesmata and functions as a potent inhibitor of molecular movement between cells^3,4^. Though PDLP5 belongs to an eight-member family of receptor-like proteins in *Arabidopsis*, it functions non-redundantly. Hence, loss of *PDLP5* alone leads to a constitutive increase in plasmodesmal permeability. Additionally, loss of *PDLP5* function results in increased susceptibility to microbial pathogens and an alteration in systemic acquired resistance^3,5^. In contrast, *PDLP5* overexpression reduces plasmodesmal opening, severely restricting overall molecular movements, and have increased immunity to pathogens^5,6^. Interestingly, systemic propagation of the secondary messenger Ca^+2^ is also deterred in *PDLP5*-overexpressor plants^7^.

The plant genome and transcriptome data that are currently available indicate that PDLPs evolved relatively recently as a ubiquitous gene family in seed plants^8^. Using confocal microscopy, correlative light and electron microscopy, and immunogold labeling, we have previously shown that PDLP5 localizes exclusively to plasmodesmata^3^. We have also shown that PDLP5 triggers plasmodesmal closure by stimulating callose synthase (CalS) 1 or CalS8 to deposit callose, a β-1,3 glucan cell wall polymer, at the plasmodesmal openings^4,9^. However, we had not yet uncovered the mechanisms behind PDLP5 plasmodesmal localization and protein activation.

**Fig. 1:**
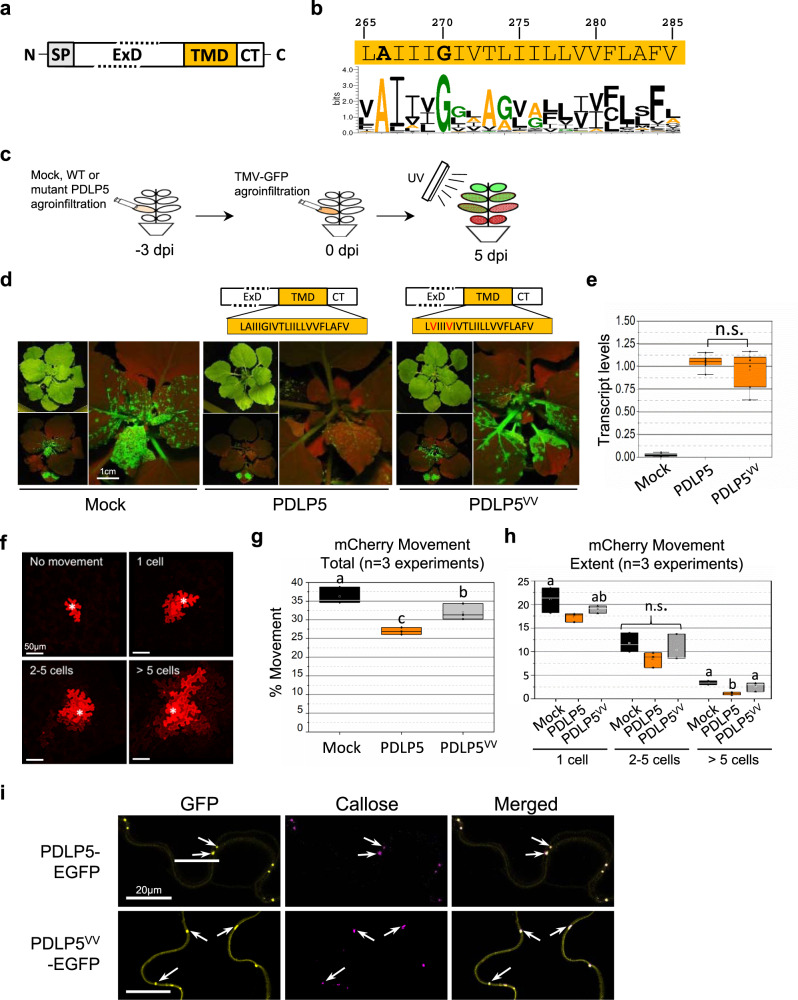
Evolutionarily conserved Ax_3_G motif in TMD is required for the PDLP5 function. **a** A receptor-like domain structure of a PDLP protein. SP, signal peptide; ExD, extracellular domain; TMD, transmembrane domain; CT, C-terminal cytosolic tail. **b** The TMD sequence of PDLP5 and a sequence logo spanning the TMD region of 491 PDLPs, generated using WebLogo. Conserved alanine and glycine residues are bold typed. **c**–**e**. TMV-GFP viral movement assays. For each movement experiment set, at least ten plants were used for mock and test treatments, and each set is repeated at least twice. A schematic illustration of the assay (**c**) and photographs of *N. benthamiana* plants exhibiting the differential impact of *PDLP5* or *PDLP5*^*VV*^, compared with mock, on the systemic movement of TMV-GFP 5-days post agroinfiltration (dpa) (**d**). The levels of PDLP5 and PDLP5^VV^ transcripts were determined by semi-quantitative RT-PCR using *N. benthamiana* leaves harvested at 3-dpa. Three biological and two technical repeats were performed per treatment. Transcript levels were normalized to that of elf1-α (**e**). Asterisks, significant differences analyzed with a one-way ANOVA followed by Fisher’s Least Significant Differences (LSD) test at the probability of *P* < 0.001 (see Source Data for details). Error bars, standard deviation. **f**–**h** Cell-to-cell movement assays using free mCherry as a reporter. Representative confocal images show the extent of the mCherry movement. Asterisks, transfected cells (**f**). Quantification of the total (**g**) and the extent (**h**) of mCherry movement. Over 550 transfected cells were examined in total using at least ten plants per treatment in three independent experimental repeats (*n* = 3). “Total” represents the percentage of the total number of cells exhibiting mCherry movement out of the total number of transfected cells (**g**), while “extent” represents the percentage of cells exhibiting movement into one or more neighboring cells out of the total number of transfected cells (**h**). Statistical analysis, a one-way ANOVA analysis followed by LSD test at the probability of P < 0.05. Letters indicate significantly different groups. Error bars, standard deviation. **i** Representative confocal images of PDLP5- or PDLP5^VV^-EGFP (false-colored in yellow) showing their localization at plasmodesmata (arrows) along the cell junctions of *N. benthamiana* epidermal cells as shown by co-localization with aniline-blue-stained callose (false-colored in magenta).

As a receptor-like, type-I transmembrane (TM) protein, PDLP5 consists of an N-terminal signal peptide, an extracellular domain (ExD) and a C-terminal TM domain (TMD) with a short cytoplasmic tail (Fig. 1a). A previous study of another PDLP member, PDLP1, experimentally validated that its C-terminal tail is exposed to the cytosol^10^. The ExD consists of two copies of the Salt stress response/antifungal domain, also known as the domain of unknown function (DUF) 26, a protein module unique to the plant kingdom. DUF26 is characterized by the presence of a conserved Cx_8_Cx_2_C motif plus three additional cysteines, together forming three intramolecular disulfide bonds. A secreted member of the DUF26 superfamily isolated from ginkgo, Gnk2, solely consists of two copies of DUF26, which  functions as a lectin that binds to mannose^11^. A recent structural study using heterologously expressed DUF26, derived from PDLP5 and PDLP8 ExD sequences, also revealed that they each form intramolecular disulfide bonds and fold similarly to Gnk2^12^.

Little is known about the function of the C-terminal domain, but the TMD of PDLP1 was shown to be indispensable for plasmodesmal localization^10^. Moreover, in the same study, the TMD alone, consisting of 21-amino acid (aa) residues, was shown to be sufficient to localize green fluorescent protein (GFP) to plasmodesmata. Whether the role of the TMD as a determinant for plasmodesmal localization is conserved among other PDLP members has remained unknown.

In the current study, we report that a specific TMD sequence of each *Arabidopsis* PDLP member is not required for the plasmodesmal targeting. Instead, it is essential for PDLPs to self-interact and anchor at the membrane. Importantly, we have found that within TMDs of virtually all predicted PDLP protein models, a dimerization motif, Ax_3_G, occurs invariably at the N-terminal side of TMDs without exception. The Ax_3_G motif is also called the Small-x-x-x-Small, where the small refers to a glycine, alanine, or serine residue^13–15^. This motif is commonly found in TMDs and known to mediate a right-handed dimerization in the membrane. We present computational modeling analyses showing that TMDs of PDLP5 and other PDLP members exhibit a high propensity to dimerize. Specifically, the prediction results show that PDLP5 TMD is able to form both right-handed and left-handed dimers mediated by multiple sets of interfacial residues, including Ax_3_G. Lastly, we discuss how the specific mode of dimerization mediated by the Ax_3_G motif might bring about PDLP5 activation and its evolutionary significance.

## Results

### PDLP5 requires a conserved Ax3G motif for its function

All eight *Arabidopsis* PDLPs are predicted to form an α-helix spanning 21-amino acid (aa) residues, according to Uniprot^16^ annotations and TMHMM^17^ and Phobius^18^ predictions. For the multiple sequence comparison, a total of 491 entries were chosen from UniProtKB for our analysis (called the PDLP reference set in Supplementary Data 1 and Supplementary Information). The taxonomic coverage of the PDLP family is restricted to seed plants (metadata in Supplementary Data 1), indicating that the protein family has evolved relatively recently. Bioinformatics analysis using multiple sequence alignment of those PDLPs revealed the presence of a few highly conserved aa residues, including alanine and glycine at the N-terminal region of TMDs. Surprisingly, these aa residues occurred invariably in all PDLP members (Fig. 1b & Supplementary Information), indicating that they must provide a yet unknown but common functionality to PDLPs.

The invariable alanine and glycine residues spaced by three variable residues formed the dimerization motif Ax_3_G. This motif is also known as the Small-x-x-x-Small motif, which frequently occurs in TM proteins and functions as an interface for interhelical interactions within the membrane^19^. Many animal cell receptors were shown to rely on this motif for dimerization and/or activation/deactivation^20–22^. To gain insight into the role of the Ax_3_G motif in PDLP5 function, we decided first to examine if alanine and glycine residues of the motif are essential for PDLP5’s activity regulating plasmodesmal opening. To this end, we constructed a PDLP5 mutant named PDLP5^VV^. This mutant carries two valine substitutions in the aa residue positions 266 and 270, which correspond to the alanine and glycine residues, respectively, of the Ax_3_G motif (Fig. 1b). Next, to evaluate whether the mutation affected the PDLP5 function, we performed systemic viral movement assays, which we had developed previously to assess the functionality of PDLP5 in a transient expression system^3^. Using this method, the extent to which the *Tobacco mosaic virus* (TMV) tagged with a GFP moves in the presence of wild-type (WT) or mutant PDLP5 is examined based on the fluorescence in systemic leaves (Fig. 1c).

As we had demonstrated before, transient overexpression of the wild-type (WT) *PDLP5* substantially delayed the systemic spread of the TMV GFP (Fig. 1d). In contrast, overexpression of *PDLP5*^*VV*^, although expressed at similar transcript levels to that of *PDLP5* (Fig. 1e), exhibited no difference to mock treatment. These results indicate that mutating the Ax_3_G motif impairs PDLP5’s activity regulating plasmodesmal opening. To further corroborate this qualitative result using a quantitative approach, we also conducted cell-to-cell movement assays using free mCherry as a reporter (Fig. 1f–h). For this, we examined a total of over 550 transfected cells per treatment in three independent experiments to record the effect of each treatment (Mock, *PDLP5*, or *PDLP5*^*VV*^) on the number of cells showing mCherry movement into neighboring cells. We categorized them into three groups: cells showing 1-cell, intermediate (2–5 cells), or extensive (>5 cells) movement.

The percentage of cells showing mCherry movement into one or more neighboring cells out of the total number of cells treated with mock, *PDLP5*, or *PDLP5*^*VV*^ was 36%, 27%, or 32%, respectively; the mean differences among treatments were statistically significant (Fig. 1g). Out of the transfected cell total, approximately 21% or 19% cells treated with Mock or *PDLP5*^*VV*^, respectively, showed mCherry movement into an immediately adjacent cell. However, 17% treated with *PDLP5* showed one cell movement, which was statistically different from the mock treatment (Fig. 1h). Although the mean differences were statistically not significant for the intermediate movement, the overall trend of each treatment’s effect was consistent. For the extensive movement category, the mean difference between the *PDLP5* and Mock or *PDLP5*^*VV*^ was statistically significant while not significant between the Mock and *PDLP5*^*VV*^: ~3% in mock/*PDLP5*^*VV*^ and 1% in *PDLP5*-treated cells, respectively (Fig. 1h). These results are consistent with those from the viral movement assays and corroborate that the AG→VV mutation compromised PDLP5’s protein activity as a plasmodesmata regulator.

Collectively, our results revealed that intact A266 and G270 residues of the putative Ax_3_G motif were essential for the PDLP5 function.

### PDLP5 self-interacts

Having found that the two core residues of the Ax_3_G motif within the TMD are crucial for the PDLP5 function, we hypothesized the possibility that those residues were imperative for a TMD-based plasmodesmal localization. However, a subsequent examination of the subcellular localization disputed this possibility. PDLP5^VV^-EGFP exhibited typical punctate plasmodesmata localization patterns co-localizing with callose similar to those of PDLP5-EGFP (Fig. 1i). Considering the role of Ax_3_G as a dimerization motif required for the activation/deactivation of various receptors^14,15,20–22^, next, we tested another possibility that A266 and G270 residues provide interfaces essential for PDLP5 to undergo a specific TMD-based self-interaction.

**Fig. 2:**
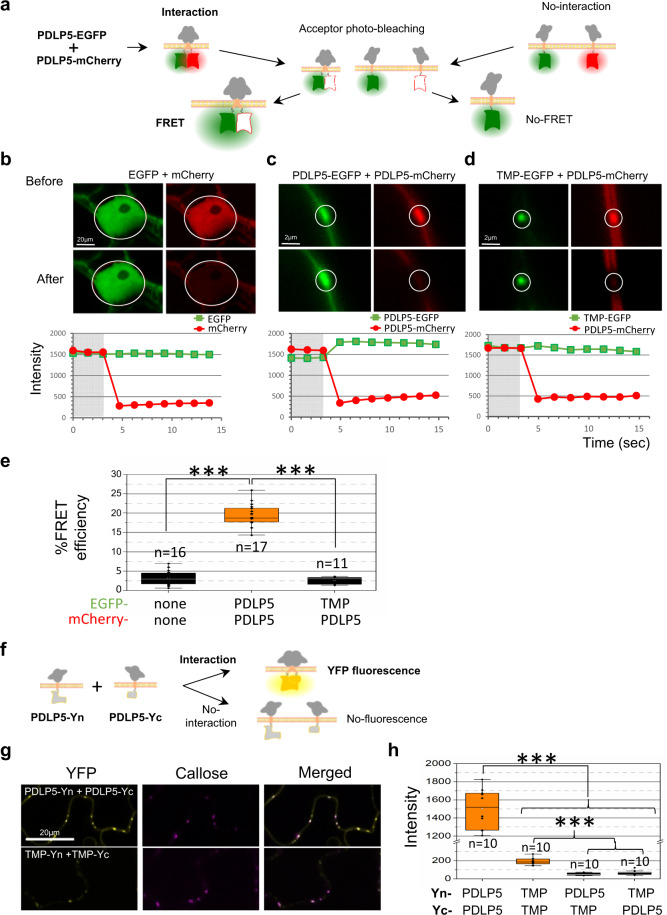
PDLP5 self-interacts via its TMD irrespective of the conserved Ax_3_G motif. **a**–**e** PDLP5 self-interaction revealed using FRET assays. A cartoon illustrating acceptor photobleaching FRET assays (**a**). Representative single-scan confocal images taken before and after photobleaching mCherry acceptor (**b**–**d**). The nucleus of the epidermis (**b**) or plasmodesmata (**c**, **d**) between epidermal cells were selected as the region of interests (ROIs, white circles) for photobleaching. Representative EGFP images before and after photobleaching show qualitative detection of FRET, while corresponding intensity graphs show quantitative changes in fluorescence within ROIs. Gray boxes, time points before photobleaching. Quantification of FRET efficiencies (**e**). No FRET occurs between the free EGFP and mCherry pair within nuclei and between the TMV MP (TMP)-EGFP and PDLP5-mCherry pair at plasmodesmata. Three asterisks represent a statistical difference at *P* < 0.001 by a Student’s *t* test. *n*, the total number of separate FRET experiments performed; n.s., no significance in statistical difference; Error bars, standard deviation. **f**–**h** PDLP5 self-interaction corroborated by BiFC assays. A schematic diagram illustrating the experimental set-up using split YFP-based BiFC of PDLP5-derived membrane protein constructs (**f**). All BiFC constructs were fused to the N-terminal (Yn) or C-terminal (Yc) half of YFP. Representative BiFC images (**g**). Confocal images were taken 30 hours post-agroinfiltration (hpa) to detect reconstituted YFP signals, which are false-colored in yellow. Plasmodesmata were discerned using callose staining and false-colored in magenta. Bars, 20 µm. Graph showing the mean intensity values of YFP signals associated with plasmodesmata (**h**). At least two plants were used to collect at least 3–5 single-scan confocal images per BiFC pair. Experiments were repeated at least twice. Fluorescence intensity was measured over individual plasmodesmata spots that were randomly selected by manually drawing region of interest over each spot using ImageJ. Statistical analyses were performed using a one-way ANOVA, followed by pairwise Tukey’s test at the probability of *P* < 0.001. *n,* the total number of YFP puncta at plasmodesmata analyzed; asterisks, significant difference; n.s., no significant difference; Error bars, standard deviation.

To examine if PDLP5 self-interacts at plasmodesmata, we performed the fluorescence resonance energy transfer (FRET) and bimolecular complementation (BiFC) assays. For FRET assays, we decided to conduct an acceptor photobleaching using combinations of EGFP (donor) and mCherry (acceptor) fusion constructs (Fig. 2a) co-expressed in *N. benthamiana* leaf epidermal cells, as we had previously described^23^. Background FRET levels were assessed using free EGFP and mCherry as a negative control by photobleaching the acceptor in the entire nuclei of epidermal cells that co-express the donor at similar levels (Fig. 2b). The FRET between PDLP5-EGFP/-mCherry pair was measured by photobleaching the acceptor at plasmodesmata between epidermal cells (Fig. 2c). In addition to the free EGFP/mCherry control, we have examined if plasmodesmal localization itself could elicit non-specific FRET to occur because of their confining nature. For this test, we evaluated if FRET occurs between an EGFP fusion of the movement protein (MP) encoded by TMV and PDLP5-mCherry (Fig. 2d). A similar FRET control experiment was previously reported using TMV MP^24^, which is not an integral membrane protein but localizes at plasmodesmata.

**Fig. 3:**
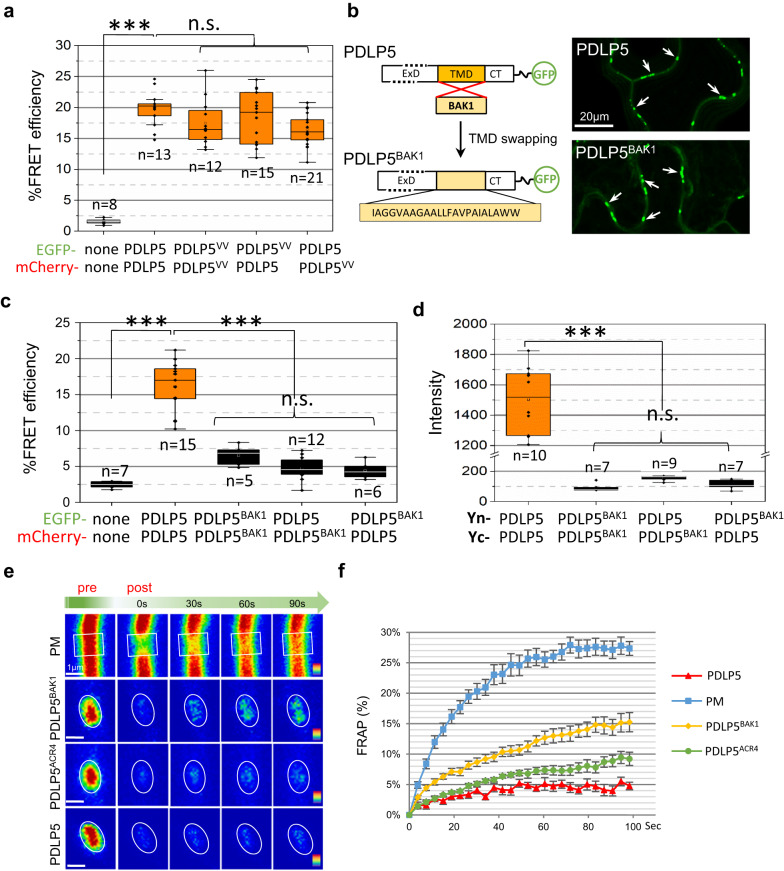
Innate TMD confers PDLP5 strong retention at plasmodesmata, not plasmodesmal targeting. **a** FRET efficiency measurements show that PDLP5^VV^ interacts with PDLP5 and PDLP5^VV^ at plasmodesmata. Asterisks represent significant differences when analyzed by a one-way ANOVA followed by Tukey’s test at the probability of *p* < 0.001. *n*, the total number of separate FRET experiments performed; n.s., no significant difference; Error bars, standard deviation. **b**–**d** PDLP5^BAK1^ having monomeric TMD localizes to plasmodesmata but does not interact with PDLP5. A diagram showing the construction of the TMD swap mutant PDLP5^BAK1^-EGFP and its plasmodesmal localization (**b**). PDLP5 and PDLP5^BAK1^ sequences are identical except for the TMD regions. Representative confocal images of PDLP5 and PDLP5^BAK1^, 15 µm-thick 3-D z-stacks, showing plasmodesmal localization (arrows). FRET (**c**) and BiFC (**d**) assays indicate that PDLP5^BAK1^ molecules form monomers and do not interact with PDLP5. Asterisks represent significant differences when analyzed using a one-way ANOVA followed by Tukey’s test at the probability of *p* < 0.001. *n*, the total number of separate FRET experiments performed (**c**) or randomly selected plasmodesmata from at least 3–5 images analyzed per BiFC pair (**d**); n.s., no significant difference; Error bars, standard deviation. **e**, **f** FRAP assays showing that PDLP5 TMD confers the protein a minimal recovery after photobleaching. Representative confocal images collected in a time series (**e**). PDLP5 and swap mutant constructs are C-terminally fused to EGFP. Images show color-coded GFP fluorescence intensities before (pre) and after (post) the onset of the photobleaching. Circles and boxes correspond to ROIs photobleached with the 488 nm Argon laser line at 100% for 1.5 s after the third scan. The results of the fluorescence recovery of each construct are plotted as % recovery over time (**f**). A total of 26-time points were recorded and plotted over a 111-second period. At least ten separate FRAP experiments were performed for each construct. Error bars, standard deviation.

As expected, no FRET occurred between free EGFP and mCherry, yielding only background levels of FRET efficiency (~5%) (Fig. 2b, e). Importantly, while TMV MP-EGFP and PDLP5-mCherry reasonably co-expressed at plasmodesmata, they did not FRET (Fig. 2d, e). Notably, another FRET experiment presented later in Fig. 3d, in which WT PDLP5 and its plasmodesmata-localizing TMD swap mutant PDLP5^BAK1^ were used as a FRET pair, revealed that they do not FRET. This result corroborates that a co-localization of two TM proteins within plasmodesmata is not in itself sufficient to generate a positive FRET signal. Finally, the FRET experiment using PDLP5-EGFP and -mCherry was performed (Fig. 2c). In contrast to negative control results, the pair yielded substantially high levels of FRET efficiency, reaching approximately 20% on average. Collectively, our FRET data indicate that the PDLP5 self-interacts at plasmodesmata.

Next, we validated PDLP5 self-interaction using a split YFP-based BiFC (Fig. 2f–h & Supplementary Fig. 1). PDLP5 was fused to the N-termini of N-terminal (Yn) and C-terminal (Yc) halves of YFP, and this BiFC pair was evaluated for positive YFP fluorescence. Co-expression of PDLP5-Yn/Yc pair showed strong, punctate YFP fluorescence at plasmodesmata co-localizing with callose (Fig. 2g, h). In contrast, BiFC pairs examined for heteromeric interactions between PDLP5 and TMV MP produced no detectable levels of YFP fluorescence (Fig. 2h & Supplementary Fig. 1). Note that TMV MP-Yn/Yc pair exhibited a substantially lower fluorescence intensity compared to PDLP5-Yn/Yc; however, the punctate YFP signals were co-localized with callose and the signal intensity was statistically higher than that from cells expressing PDLP5/TMV MP heteromeric BiFC pairs. Collectively, these results provide strong experimental evidence that PDLP5 self-interacts at plasmodesmata and this interaction is highly specific.

### PDLP5 self-interacts via TMD irrespective of the Ax3G motif

Next, we examined whether the Ax_3_G motif in PDLP5 TMD is required for the PDLP5 self-interaction, again using FRET and BiFC. The results showed that PDLP5^VV^ could form not only homomers but also heteromers with PDLP5 (Fig. 3a and Supplementary Fig. 2). Based on this result, we concluded that the Ax_3_G motif was not required for PDLP5 self-interaction. However, puzzled by the result showing the dispensability of Ax_3_G for self-interaction, we decided to investigate if PDLP5 self-interaction is mediated through the TMD or ExD.

Since the TMD is essential for the membrane anchoring of PDLP5 (Supplementary Fig. 3), we pursued a domain swapping approach, in which the PDLP5 TMD was replaced with a foreign α-helical TMD derived from a monomeric PM protein. For this, we chose the TMD of the RLK BAK1 as a suitable sequence because the BAK1 TMD was experimentally shown to be monomeric in a similar domain swapping study^25^. We reasoned that if PDLP5 self-interacts through its TMD, swapping its native TMD with that of BAK1 would cause the resulting mutant (PDLP5^BAK1^) to be monomeric. Notably, different bioinformatics tools predicted the BAK1 TMD slightly differently in lengths (e.g., 21 or 23 residues) and positions. For our experiment, we chose the BAK1 TMD sequence consisting of 23 aa residues (Fig. 3b), adopting the prediction output using the TMHMM server. Our experiments, described later in the current study, using computational modeling to predict TMD dimerization indicated that either the 23mer BAK1 TMD sequence we used or 21mer predicted by other programs or even longer sequences all to be monomeric.

Confocal imaging of PDLP5^BAK1^-EGFP showed that it localized to plasmodesmata (Fig. 3b), as demonstrated by callose co-localization (Supplementary Fig. 4). This result indicated that the specific PDLP5 TMD sequence was not required to localize at plasmodesmata, allowing us to examine if PDLP5^BAK1^ self-interacts or not via the ExD. Subsequent FRET experiments showed that PDLP5^BAK1^-EGFP/–mCherry pair did not FRET, demonstrating that the ExD did not mediate PDLP5 self-interaction (Fig. 3c). Besides, no FRET occurred between PDLP5 and PDLP5^BAK1^, either. Furthermore, BiFC experiments also supported PDLP5^BAK1^ being monomeric (Fig. 3d and Supplementary Fig. 5). The significance of these findings is twofold: (1) these results corroborate that PDLP5 self-interaction is mediated through its native TMD; (2) the specific sequence of the TMD is not required for the plasmodesmal localization of PDLP5.

### Native TMD is required to immobilize PDLP5 at plasmodesmata

Having found that self-interacting or non-interacting TMD both similarly localize at plasmodesmata, we asked if their mobilities were also similar. To gain insight into this question, we compared the diffusion rates of PDLP5 and PDLP5^BAK1^ in fluorescence recovery after photobleaching (FRAP) experiments (Fig. 3e, f). As a control, a derivative of the PM-localized RLK BAK1 was constructed as an EGFP fusion (PM-EGFP). Subsequent FRAP assays showed that the PM-EGFP yielded approximately 25% fluorescence recovery in less than 50 s after photobleaching. This recovery rate was comparable to those of PM-localizing plant TM proteins shown by FRAP^26^.

Next, additional FRAP experiments were performed over plasmodesmata signals produced by PDLP5- or PDLP5^BAK1^-EGFP at the epidermal-epidermal cell junctions (Fig. 3e, f). These experiments showed that PDLP5^BAK1^ exhibited a 15% recovery within less than 80 s, which denotes that restricting the protein at the plasmodesmata markedly reduced its mobility as compared with localizing a protein at the PM. Surprisingly, PDLP5 was immobile at the plasmodesmata; it showed no more than 5% recovery even 100 s after photobleaching. This result suggests that PDLP5 likely forms a large, stable complex at plasmodesmata. Intrigued by this result, we produced another TMD swap mutant, PDLP5^ACR4^, containing the TMD derived from the RLK ACR4. ACR4 is a PM-localized RLK that can partially associate with plasmodesmata via its oligomerizing-TMD^25^. As expected, confocal imaging showed that PDLP5^ACR4^ also localized at plasmodesmata (Supplementary Fig. 6; also see Fig. 5c). However, FRAP measurements revealed an intermediary fluorescence recovery between PDLP5 and PDLP5^BAK1^. Although there might be other explanations for the differential mobility shown by these TMD swap mutants, our results seem to suggest that the PDLP5 TMD likely confers a particular mechanism to immobilize the protein at plasmodesmata (Fig. 3e, f).

### TMD-sequence or -length does not determine PDLP localization

**Fig. 4:**
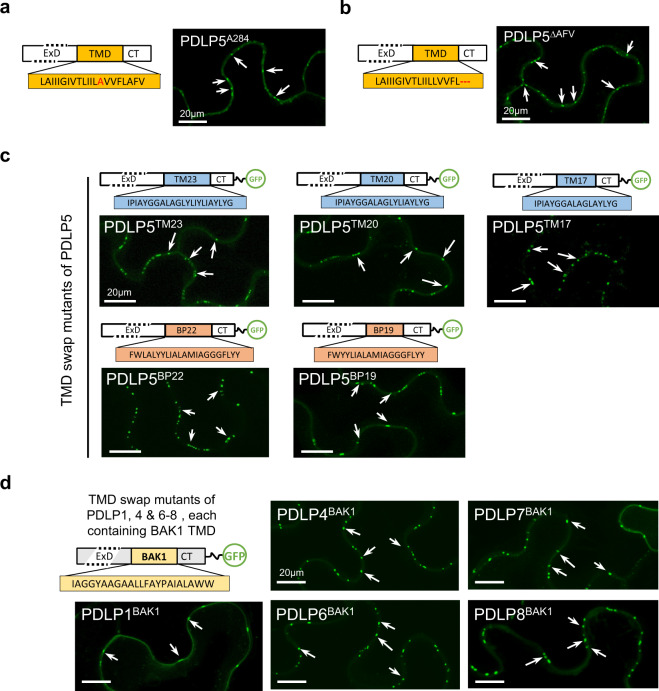
Plasmodesmal localization is independent of TMD amino acid composition or length. Schematic diagrams illustrating various PDLP5 TMD mutants, with representative confocal images showing their plasmodesmal localization. Alanine mutation of F284 (**a**) or a deletion mutation lacking three C-terminal amino acid residues (**b**)  of the TMD does not affect PDLP5 localization. A schematic diagram illustrating PDLP5 TMD swap mutants, TM23, TM20, TM17, BP22, and BP19, with representative confocal images showing plasmodesmal localization (See also Supplementary Fig. 6) (**c**). A schematic diagram illustrating PDLP1, 4, 6–8 TMD swap mutants containing the BAK1 TMD, with representative confocal images showing plasmodesmal localization (See also Supplementary Fig. 7).

Previously, a subcellular localization study of PDLP1 had identified a valine residue within the TMD to be a crucial factor determining the plasmodesmal targeting of the protein. The same study had also reported that keeping the TMD length no shorter than 21mer was critical^10^. The aa residue corresponding to that valine is F284 in PDLP5. To evaluate if this residue had similar functional importance, we constructed a GFP-tagged alanine substitution mutant, PDLP5^A284^-EGFP. A subsequent localization study, however, revealed that PDLP5^A284^-EGFP localized to plasmodesmata normally (Fig. 4a). Moreover, neither the shortening of the PDLP5 TMD from 21mer to 18mer had any impact on PDLP5 localization (Fig. 4b). These results first seemed to suggest that PDLP5 could have different requirements of the TMD for its targeting.

To further corroborate on that point, we produced additional TMD swap mutants, in which the PDLP5 TMD was replaced with other foreign TMDs varying in both sequences and lengths. For this, five TMD sequences were chosen, namely, TM23 and BP22 and their shorter derivatives, TM20, TM17, and BP19^27^. TM23 and BP22 are PM-localizing 23- and 22-aa-long TMDs, respectively; their derivatives shortened in TMD lengths by three or more aa residues are retained at Golgi (TM20 & BP19) or ER (TM17). Surprisingly, these TMD swap mutants all showed normal plasmodesmal localization. These results validate that PDLP5 localization is independent of TMD lengths and composition (Fig. 4c and Supplementary Fig. 6).

Next, we examined if PDLP5, or perhaps even other PDLP members yet unstudied, have different requirements of the TMD from PDLP1 for plasmodesmal localization. For this, we constructed TMD swap mutants of PDLPs that are more closely (PDLP6, PDLP7, and PDLP8) or remotely (PDLP4) related to PDLP5, including PDLP1, by replacing their TMDs with BAK1 TMD. Confocal imaging showed that these swap mutants also all localized normally at plasmodesmata (Fig. 4d & Supplementary Fig. 7).

Based on these results, we conclude that PDLP5 is not unique in having no specific requirements of the TMD for its targeting, but other PDLP members share this characteristic.

### PDLP5 TMD exhibits a high propensity to dimerize

**Fig. 5:**
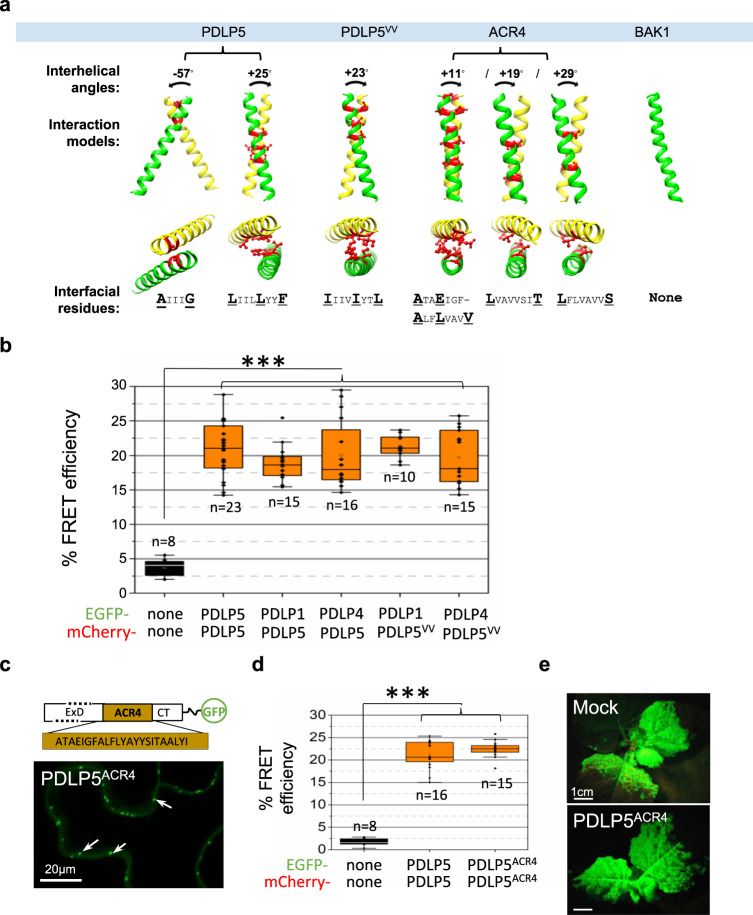
PDLP5 TMD shows a high propensity to dimerize. **a** Homomeric dimerization models predicted using TMDOCK and visualized using GLmol. Note that BAK1 TMD is predicted to form a monomer. Right- or left-handedness of dimers is represented by a counterclockwise or clockwise black arrow and negative or positive signs, respectively. Predicted interfacial residues are colored in red in each model, and bold typed in each motif sequence. **b** Heteromeric FRET occurs between PDLP5 and PDLP1 or PDLP4 and PDLP5^VV^ and PDLP1 or PDLP4. Asterisks represent significant differences when analyzed using a one-way ANOVA followed by Tukey’s test at the probability of *P* < 0.001. *n*, the total number of separate FRET experiments performed; n.s., no significant difference; Error bars, standard deviation. **c–e** Localization, self-interaction, and viral movement assays performed using PDLP5^ACR4^. ACR4 TMD can substitute PDLP5 TMD for localization (**c**) and self-interaction (**d**), but not plasmodesmata-regulating function (**e**). Asterisks represent significant differences when analyzed using a one-way ANOVA followed by Tukey’s test at the probability of *P* < 0.001. *n*, the total number of separate FRET experiments performed; Error bars, standard deviation.

As described earlier, we have shown that the motif Ax_3_G is not required for the TMD-mediated self-interaction. This data led us to suspect the presence of an alternative dimerization motif(s) in PDLP5 TMD. To gain more insight into this question, we turned our attention to computational predictions, utilizing TMDOCK and PREDDIMER programs. Notably, both programs predicted the BAK1 TMD sequence to be monomeric (Fig. 5a), regardless of whether the TMD sequence analyzed included just the core 21mer or was extended to some 30mers. It is noteworthy that BAK1 TMD contains an Ax_3_G or similar motifs at the N-terminal side. However, the occurrence of these motifs did not affect the prediction. Also, experimentally, an extracellularly extended BAK1 TMD, including the putative Ax_3_G motif, was shown to be monomeric by Stahl et al^25^.

In contrast, the PDLP5 TMD sequence was predicted to exhibit a high propensity to form both right- and left-handed dimers. According to the prediction results, various small and hydrophobic aa residues, including Ax_3_G and leucine heptad-like (Lx_3_Lx_2_F) motifs, could all contribute to the dimer formation (Fig. 5a and Supplementary Data 2). Notably, the Ax_3_G motif allowed a very stable right-handed homomeric dimer to form, which was lacking in PDLP5^VV^ TMD (Supplementary Data 2). However, the absence of Ax_3_G did not abolish dimer formation as PDLP5^VV^ TMD could still produce a stable left-handed dimer according to the prediction (Fig. 5a and Supplementary Table1). This prediction result is consistent with our positive FRET and BiFC data.

The high propensity to dimerize was also exhibited by TMDs of other PDLP family members (Supplementary Data 2). Not surprisingly, one dimer model shared by all eight PDLP members was derived from the invariable Ax_3_G motif. Because the Ax_3_G motif was highly conserved among PDLPs, we reasoned that if they formed heteromeric complexes, it would be most likely mediated by this motif. Therefore, to gain an insight into the role of Ax_3_G for dimerization, we examined if PDLP5 would interact with other PDLP members. For heteromeric FRET experiments, we chose PDLP1 and PDLP4 as FRET partners for PDLP5 because these two members were more remotely related paralogs to PDLP5, and their TMDs were dissimilar in aa residue composition. As expected, FRET occurred between PDLP5 and PDLP1 or PDLP4, yielding high levels of the FRET efficiency (Fig. 5b). We then tested if PDLP5^VV^ interacts with PDLP1 or PDLP4. We anticipated that if the conserved motif Ax_3_G was required for the heteromeric interactions, PDLP5^VV^ would not FRET with other PDLPs. However, the result showed that PDLP5^VV^ FRETs with PDLP1 or PDLP4 (Fig. 5b). These FRET results were also confirmed by BiFC data (Supplementary Fig. 8). Molecular modeling using the PREDDIMER program also showed that PDLP5 or PDLP^VV^TMD could form heterodimers with PDLP1 or PDLP4 TMD. From these results, we concluded that the Ax_3_G motif was not required for heteromeric interactions.

### A specific TMD dimerization is required for PDLP5 activity

The high propensity to dimerize, predicted for the TMD of PDLP5 and other members, is consistent with our experimental data showing that the elimination of Ax_3_G is not sufficient to impair TMD-based interactions. On the one hand, the prediction that the Ax_3_G-based right-handed model is most stable seems to explain why PDLP5^VV^ lost the activity. On the other hand, the high propensity would seem to make it difficult, if not impossible, to demonstrate the role of Ax_3_G-based right-handed dimerization experimentally. Nevertheless, the prediction that PDLP5^VV^ could only form a left-handed dimer led us to ask whether PDLP5 activation might require a flexible dimerization, a capability to shift between the right-handed and left-handed configuration. This property might be necessary if the protein has to accommodate the conformational changes imposed by the ExD, the cytosolic tail, or by interactions with other proteins. In this view, we might assume that PDLP5^VV^ was impaired in plasmodesmal regulating function because it lost the capacity to form a dimer that has to be flexible to undergo shifts between left- and right-handed modes.

In an attempt to test our notion, we analyzed the dimerization predictions for the TMD domains we utilized in swap mutation studies. We searched for the TMD that closely resembled the dimerization model predicted for PDLP5^VV^. Among those, we found that ACR4 TMD was predicted to form predominantly left-handed models (Fig. 5a). First, to validate that it self-interacts, we performed FRET analysis using the PDLP5^ACR4^-EGFP/-mCherry pair, which localizes at plasmodesmata (Fig. 5c). As anticipated, PDLP5^ACR4^ self-interacted, exhibiting high levels of FRET efficiency (Fig. 5d). The BiFC experiment also confirmed the FRET result (Supplementary Fig. 9). Next, we performed a functional test using PDLP5^ACR4^ in viral movement assays. This experiment showed that similar to PDLP5^VV^ treatment, PDLP5^ACR4^ could not deter the systemic movement of TMV GFP (Fig. 5e). These results indicate that although ACR4 TMD-mediated self-interactions, it could not substitute the PDLP5 TMD for plasmodesmata-regulating function. We posit that perhaps PDLP5 requires a very particular mode of dimerization mediated by its native TMD.

Collectively, our current study provides new insights into the mechanism of PDLP5 activation and the role of its TMD. Our findings show that the TMD mediates self-interactions and consists of a functionally essential motif, Ax_3_G. Given the evolutionary conservation of this motif in all PDLPs, we anticipate that other PDLP members likely share the Ax_3_G-dependent activation mechanism described for PDLP5.

## Discussion

In our current study, we discovered that the Ax_3_G motif located at the N-terminal side of TMD was vital for the PDLP5’s protein function as a plasmodesmal regulator. This motif occurs in all predicted PDLP members, which seems to suggest that it likely provides a crucial role in the activation of other PDLPs as well. Furthermore, our fluorescence-based studies, using FRET, FRAP, and BiFC techniques, together with systemic viral movement assays, revealed that the native TMD was essential for PDLP5 to retain and self-interact at plasmodesmata as well as to regulate cell-to-cell movement.

We have also found that the PDLP5 TMD sequence was replaceable with different TMD sequences without affecting plasmodesmal localization. Similarly, TMDs of other PDLP members, including PDLP1, were also replaceable. A previous report implicated that the PDLP1 TMD sequence alone was a crucial factor determining plasmodesmal localization. According to that study, the 21mer PDLP1 TMD alone was reported to be sufficient to target a GFP variant to plasmodesmata. Moreover, the study also showed that the TMD length itself was crucial for PDLP1’s plasmodesmal localization^10^. Note that one apparent difference between our research from the previous one is that we employed substitution mutagenesis instead of deletion. Nevertheless, our current study revealed that the plasmodesmal localization of PDLP5 or other PDLP members was independent of the TMD length or sequence. Most importantly, our study disclosed that a major functional attribute of PDLP TMDs is their capacity to mediate homo- and heteromeric interactions of PDLPs.

A further investigation would be needed in the future to gain deeper insight into the role of TMD-mediated interactions for the mechanism of PDLP function and mobility. However, our data offer some hints. Firstly, our FRAP data indicate that different TMD sequences could differ in constraining protein mobility at plasmodesmata. While PDLP5 protein was immobile at plasmodesmata, its TMD swap mutant PDLP5^BAK1^ exhibited substantially higher mobility. Their TMD sequence might differ in other unknown characteristics, but they distinctively differ in their capability to form homomers. We posit that specific TMD-mediated protein interactions likely confer PDLP5 protein the stability, thereby inhibiting its lateral diffusion. This immobility exhibited by PDLP5 also suggests the possibility that its native TMD facilitates the formation of higher-order oligomers. Secondly, a specific TMD sequence composition or length is not required for plasmodesmal localization. We suppose that the plasmodesmal outer membrane, e.g., the PM leaflet, might be quite flexible or dynamic. A recent bioinformatics study supported the role of TMDs as membrane sorting determinants and showed that intracellular membrane localization of single-pass TM proteins could be predicted from the length and amino acid sequence compositions of their TMDs^28^. How the membrane composition of plasmodesmata differs from intracellular membranes in accommodating various types of TMDs is one of the questions that we are actively investigating.

The Ax_3_G motif is a variation of the Small-x-x-x-Small motif in which the “small” residues represent alanine, glycine or serine. This motif is crucial for many protein functions in animals, including TM protein dimerization, localization, protein folding and assembly, or lipid binding^29–32^. As for plant proteins, although approximately 14% of total TMDs encoded by the *Arabidopsis* proteome are suggested to contain the Small-x-x-x-Small motif^15^, to our knowledge, hardly any information is available about their functional roles in plant proteomes. Our finding that the Ax_3_G motif occurs not only in all PDLP members but also in a fixed position within their TMDs without exception suggests that this motif has evolved to play a specific function of the protein family. Given that the Ax_3_G only represents one variation of the highly prevalent Small-x-x-x-Small motif, such stark conservation may imply that the motif functions as a mechanical component critical for the protein activation, as demonstrated for PDLP5.

Our FRET/BiFC results showed that PDLP5^VV^, which is mutated to eliminate the Ax_3_G motif, maintains homo- and heteromeric interactions as well as subcellular localization. This data indicates that the motif is required not for the protein targeting nor TMD-mediated self-interactions. What then is the exact molecular function of Ax_3_G that makes it so crucial for PDLP5 activity? Computationally, PDLP5 TMD shows a high propensity to dimerize, being capable of forming both right- and left-handed dimers. Hence, alteration of the motif does not necessarily affect interhelical interactions in PDLP5^VV^ because other TMD aa residues become available to provide alternative interhelical interactions. As for the free energy requirement, though, Ax_3_G-based right-handed dimer is predicted to be most stable among various dimer models. These TMD dimer model results seem to suggest that the motif has a role in delivering a specific mode of TMD dimerization instead of being the sole motif necessary for the TMD-based interaction of PDLP5. In this view, we speculate that the role of Ax_3_G could be to facilitate PDLP5 to form a homomeric complex that is functionally active.

Apart from the computational predictions, a different role ascribable to the Ax_3_G motif might be its potential function in interacting with a downstream effector(s). Such interaction may be possible through a specific conformation of PDLP5 that is facilitated directly by Ax_3_G-dependent self-interaction or indirectly by Ax_3_G-mediated interactions with other effectors. Given that PDLP5 regulates plasmodesmal permeability not by itself but by elevating callose levels, a prime candidate for such effector would be plasmodesmata-specific callose synthases. In our previous study using double and triple mutants, we had shown that PDLP5 requires specific CalS: CalS8 to maintain basal levels of plasmodesmal opening and CalS1 during immune responses to restrict molecular transport systemically as well as locally^9^. In that study, we had suggested that PDLP5 and CalS1/8 most likely work together at the level of a protein complex. If they form a complex, PDLP5 may directly interact with both CalS1 and CalS8, or it may interact with a yet-unknown intermediate protein. In either scenario, Ax_3_G could provide a key motif necessary for specific interactions or allow the PDLP5 conformation compatible with such molecular interactions.

In addition to Ax_3_G-based right-handed dimers, PDLP TMDs are predicted to form left-handed dimers using various combinations of hydrophobic heptad residues occurring along the length of their TMDs. What could be the functional significance of this high propensity to dimerize? One tantalizing answer to this question is that the flexible dimerization might serve as a mechanism for PDLP to switch between active and inactive forms. Alternatively, a shift between right- and left-handed dimeric configurations may be necessary for PDLPs to form a versatile signaling complex with RLPs or RLKs. Or, the high propensity to dimerize may enable PDLPs to attract specific integral or peripheral membrane proteins to plasmodesmata. These proteins may associate, transiently, or stably, with PDLPs via TMD-mediated interactions.

In summary, our present study highlights the critical roles of the TMD for PDLP function, which warrants further investigations in the future at the molecular and protein structural levels.

## Methods

### Plasmid cloning and transient expression

For the construction of FP fusion and chimeric proteins, overlapping PCR^33^ was performed using the Phusion high-fidelity DNA polymerase (New England Biolabs) to amplify DNA inserts, followed by restriction enzyme digestion and ligation using the T4 DNA ligase (New England Biolabs). For cloning, we utilized our in-house binary vector pMB35S, designed to allow directional cloning using the restriction enzyme SfiI^34^. Briefly, pMB35S is derived from a Gateway binary vector, dpGreen-BarT, replacing the Gateway component with an expression cassette comprised of the 35S promoter, multicloning sites, and the 19S terminator. The 2-kb-long Gateway component was removed from the parent plasmid using *Kpn*I and *Sac*I double restriction enzyme digestion. The GFP, mCherry, or half-YFP fusion constructs were cloned into pMB35S using two *Sfi*I sites to allow for directional cloning from single restriction enzyme digestion. A glycine linker sequence (ggccggcctggaggtggaggtggaccg) was inserted between the 3′-end of *Sfi*I site following the C-terminal residue of a protein of interest and the N-terminus of an FP. All primers used for cloning and RT-PCR are provided in Supplementary Data 3. The fidelity of each construct was confirmed by Sanger sequencing. WT and mutant fusion constructs were transformed into *Agrobacterium tumefaciens*, strain GV3101 (+pSoup), for transient expression in mature leaves of 4-week old *N. benthamiana* plants. For subcellular localization and FRET assays, the concentration of resuspended agrobacteria was adjusted to OD_600_ = 0.5. For BiFC experiments, samples were microscopically examined 30-hours post agroinfiltration (hpa).

### Bioinformatics TMD sequence analysis

Details on the sequence retrieval of PDLP family members from UniProt (reference set) is described in Supplementary Note. The TMD sequences of PDLP were predicted using TMHMM 2.0 (http://www.cbs.dtu.dk/services/TMHMM/), and multiple sequence alignment was performed using CLUSTAL (https://www.ebi.ac.uk/Tools/msa/). The sequence logo spanning TMD of PDLP was produced using a WebLogo generator with custom colors orange for A and green for G^35^. An alignment of the TMD region in PDLP and adjacent aa (corresponding to region 264–303 in PDLP1, Q8GXV7) was used as a query in an HMMER search (HmmerWeb version 2.35.0^36^,) against UniProt Reference Proteome set to see if the HMM model for this region could be sufficient to retrieve PDLP homologs. This search efficiently retrieved the PDLP family members (over 90% of entries) when compared to the UniProt search reference set.

### FRET, BiFC, and FRAP analyses

Acceptor photobleaching FRET analysis was carried out as described previously^23^. Ten multi-scanned images for both free or fused EGFP and mCherry proteins were collected under donor excitation wavelength (488 nm) and acceptor excitation wavelength (561 nm), respectively. Images were acquired using the laser power set at 0.2% maximum to minimize photobleaching. The mCherry was photobleached by continuously scanning region of interest (ROI) with the 561-nm laser line set at 100% intensity for 10 s after the third scan. Recovery of the donor from quenching was quantified by subtracting the post-bleaching donor emission from that of pre-bleaching. The FRET efficiencies were calculated after automatic background subtraction from manually defined background ROIs. Processing and analysis of FRET images, the pseudo-coloring of fluorescence intensities, and calculation of FRET efficiencies were performed using the software and protocols of Zeiss Zen 2010 D (v7.0.0.223).

For BiFC experiments, each pair was transiently expressed by agroinfiltration in which *N. benthamiana* leaves were infiltrated with a 1:1 mixture of *A. tumefaciens* strain GV3101 (+pSOUP) carrying pMB35S plasmids containing half-YFP fusion constructs. Each transformed GV3101 colony was cultured in the liquid until the OD600nm reached 0.4. Transfected cells expressing each BiFC pair were microscopically analyzed 29- to 31-hpa.

For each FRAP experiment, thirty continuously scanned images were collected within an average of 111 s time series. The ROI was photobleached with the 488 nm Argon laser line at 100% for 1.5 s after the third scan. At least a 50% fluorescence intensity reduction was obtained from quenching. FRAP recovery rates were calculated by subtracting the fluorescence intensity at the last scan from the fluorescence intensity at the first post photobleaching scan (the fourth scan) and divided by the initial fluorescence intensity. At least ten individual ROIs were collected for each fluorescent protein, and the result was plotted using average FRAP recovery rate at each data point. To avoid drifting while imaging, plant tissues were cut into 5 mm × 5 mm square pieces and mounted in water in an imaging chamber. Imaging was performed under conditions with minimal lighting and room temperature no higher than 20 °C.

### Confocal microscopy and image acquisition

Subcellular localization, FRET and BiFC images were acquired using LSM 880 confocal microscope while FRAP images LSM 710 META, each equipped with an inverted scan head Plant samples were placed into single well Lab-Tek®II Chambers, covered with coverslips and flattened gently with a glass weight. The GFP tagged fusion proteins were imaged using a C-Apochromat X40/1.20-W Korr UV–VIS–IR objective with the 488-nm Argon laser and 505–550 nm band-pass emission filter. The mCherry tagged fusion proteins were using the same objective with the 561-nm DPSS laser and 575–615 nm band-pass emission filter. The YFP fluorescence was detected using 514 nm excitation line and 517–579 nm emission channel. Aniline-blue-stained callose imaging was performed on a Zeiss LSM880, using a C-Apochromat X40/1.20-W Korr UV–VIS–IR objective with the 405-nm Diode laser and a 420–480 nm band-pass emission filter. The chloroplast autofluorescence was detected through a 636–754 nm emission filter. For representative localization images, a series of optical sections was acquired as Z-stacks and rendered as 3-D projection with Zeiss LSM Image Examiner or Zen software or with ImageJ. Images of viral movement assays were taken by Nikon D3100 DSLR camera with 18–55 mm f/3.5–5.6 auto Focus-S Nikkor Zoom lens. A deep-yellow-15 lens filter (TIFFEN, 52 mm) was used for GFP visualization.

### Movement assays

To perform viral movement assays, two mature leaves of each ten *N. benthamiana* plants were infiltrated with mixed agrobacterial cells that carry genes encoding full-length WT PDLP5 or mutant construct and the viral suppressor protein p19. Three days after this primary agroinfiltration, the same leaves were re-infiltrated with agrobacterial cells carrying a TMV-GFP (OD_600_ = 0.2). Five days after the secondary agroinfiltration, the extent to which the systemic movement of the virus occurred was recorded by photographing the plants under a UV illumination through a deep-yellow filter mounted on a Nikon D3100 digital camera. mCherry protein movement assays were performed similarly except that the secondary infiltration was done with agrobacteria carrying free mCherry (pMB:35S-mCherry) diluted to 1 × 10^4^ cells/ml to disperse transfection events sufficiently far from each other. Recording of the extent to which mCherry movement occurred out of the transfected individual cells was performed under a fluorescence microscope (Zeiss Axiovert 200).

### TMD modeling

Prediction of TMD dimerization modes for PDLPs and PDLP5 derivatives was performed using two web servers, TMDOCK (http://membranome.org/) and PREDDIMER (http://model.nmr.ru/preddimer/) following the instructions and information provided by those servers. 3D dimer models were downloaded as PDB files and visualized using GLMol (https://webglmol.osdn.jp) or UCSF Chimera (https://www.cgl.ucsf.edu/chimera/).

### Statistics and reproducibility

Statistical analyses and dot/box plots were created using Origin 2020 (OriginLab) software. Statistical methods and probability values used to analyze relevant experiments are described in the figure legends. Each experiment requiring statistical analysis was performed at least three times and was reproducible.

### Reporting summary

Further information on research design is available in the Nature Research Reporting Summary linked to this article.

## Supplementary information

Supplementary Information

Description of Additional Supplementary Files

Supplementary Data 1

Supplementary Data 2

Supplementary Data 3

Supplementary Data 4

Reporting Summary

## Data Availability

Proteins discussed in this work correspond to the following UniProtKB database entries: PDLP1 (Q8GXV7), PDLP2 (Q6NM73), PDLP3 (O22784), PDLP4 (Q6E263), PDLP5 (Q8GUJ2), PDLP6 (Q9ZU94), PDLP7 (Q0WPN8), PDLP8 (Q6NKQ9), BAK1 (Q94F62), ACR4 (Q9LX29). All source data associated with graphs presented in Figs. 1e, g, h, 2e, 2h, 3a, c, d, 5b, and d, and Supplementary Fig. 1b are provided in Supplementary Data 4. All other data that support the findings of this study are available from the corresponding author upon reasonable request.

## References

[1] Liu L, Chen X (2018). Intercellular and systemic trafficking of RNAs in plants. Nat. Plants.

[2] Gallagher KL, Sozzani R, Lee CM (2014). Intercellular protein movement: deciphering the language of development. Annu. Rev. Cell Dev. Biol..

[3] Lee JY (2011). A plasmodesmata-localized protein mediates crosstalk between cell-to-cell communication and innate immunity in Arabidopsis. Plant Cell.

[4] Wang X (2013). Salicylic acid regulates plasmodesmata closure during innate immune responses in Arabidopsis. Plant Cell.

[5] Lim GH (2016). Plasmodesmata localizing proteins regulate transport and signaling during systemic acquired immunity in plants. Cell Host Microbe.

[6] Carella P, Isaacs M, Cameron RK (2015). Plasmodesmata-located protein overexpression negatively impacts the manifestation of systemic acquired resistance and the long-distance movement of Defective in Induced Resistance1 in Arabidopsis. Plant Biol. (Stuttg.).

[7] Toyota M (2018). Glutamate triggers long-distance, calcium-based plant defense signaling. Science.

[8] Lee JY (2014). New and old roles of plasmodesmata in immunity and parallels to tunneling nanotubes. Plant Sci.: Int. J. Exp. plant Biol..

[9] Cui W. & Lee J. Y. Arabidopsis callose synthases CalS1/8 regulate plasmodesmal permeability during stress. *Nature Plants* **2**, 16034 (2016). 10.1038/nplants.2016.34 27243643

[10] Thomas CL, Bayer EM, Ritzenthaler C, Fernandez-Calvino L, Maule AJ (2008). Specific targeting of a plasmodesmal protein affecting cell-to-cell communication. PLoS Biol..

[11] Miyakawa T (2014). A secreted protein with plant-specific cysteine-rich motif functions as a mannose-binding lectin that exhibits antifungal activity. Plant Physiol..

[12] Vaattovaara A (2019). Mechanistic insights into the evolution of DUF26-containing proteins in land plants. Commun. Biol..

[13] Li E, Wimley WC, Hristova K (2012). Transmembrane helix dimerization: beyond the search for sequence motifs. Biochim. Biophys. Acta.

[14] Lock A (2014). One motif to bind them: A small-XXX-small motif affects transmembrane domain 1 oligomerization, function, localization, and cross-talk between two yeast GPCRs. Biochim. Biophys. Acta.

[15] Teese MG, Langosch D (2015). Role of GxxxG motifs in transmembrane domain interactions. Biochemistry.

[16] UniProt C (2019). UniProt: a worldwide hub of protein knowledge. Nucleic Acids Res..

[17] Sonnhammer EL, von Heijne G, Krogh A (1998). A hidden Markov model for predicting transmembrane helices in protein sequences. Proc. Int Conf. Intell. Syst. Mol. Biol..

[18] Kall L, Krogh A, Sonnhammer EL (2004). A combined transmembrane topology and signal peptide prediction method. J. Mol. Biol..

[19] Moore DT, Berger BW, DeGrado WF (2008). Protein-protein interactions in the membrane: sequence, structural, and biological motifs. Structure.

[20] Bugge K., Lindorff-Larsen K., & Kragelund B. B. Understanding single-pass transmembrane receptor signaling from a structural viewpoint-what are we missing? *FEBS J.* **283**, 4424–4451 (2016). 10.1111/febs.13793 27350538

[21] Walther TH, Ulrich AS (2014). Transmembrane helix assembly and the role of salt bridges. Curr. Opin. Struct. Biol..

[22] Zhou FX, Cocco MJ, Russ WP, Brunger AT, Engelman DM (2000). Interhelical hydrogen bonding drives strong interactions in membrane proteins. Nat. Struct. Biol..

[23] Zhou J, Wang X, Lee JY, Lee JY (2013). Cell-to-cell movement of two interacting AT-hook factors in Arabidopsis root vascular tissue patterning. Plant Cell.

[24] Amari K, Lerich A, Schmitt-Keichinger C, Dolja VV, Ritzenthaler C (2011). Tubule-guided cell-to-cell movement of a plant virus requires class XI myosin motors. PLoS Pathog..

[25] Stahl Y (2013). Moderation of Arabidopsis root stemness by CLAVATA1 and ARABIDOPSIS CRINKLY4 receptor kinase complexes. Curr. Biol..

[26] Martiniere A (2012). Cell wall constrains lateral diffusion of plant plasma-membrane proteins. Proc. Natl Acad. Sci. USA.

[27] Brandizzi F (2002). The destination for single-pass membrane proteins is influenced markedly by the length of the hydrophobic domain. Plant Cell Online.

[28] Sharpe HJ, Stevens TJ, Munro S (2010). A comprehensive comparison of transmembrane domains reveals organelle-specific properties. Cell.

[29] Dixon AM (2014). Differential transmembrane domain GXXXG motif pairing impacts major histocompatibility complex (MHC) class II structure. J. Biol. Chem..

[30] Marinangeli C (2015). Presenilin transmembrane domain 8 conserved AXXXAXXXG motifs are required for the activity of the gamma-secretase complex. J. Biol. Chem..

[31] Bronnimann MP, Chapman JA, Park CK, Campos SK (2013). A transmembrane domain and GxxxG motifs within L2 are essential for papillomavirus infection. J. Virol..

[32] Barrett PJ (2012). The amyloid precursor protein has a flexible transmembrane domain and binds cholesterol. Science.

[33] Shevchuk NA (2004). Construction of long DNA molecules using long PCR-based fusion of several fragments simultaneously. Nucleic Acids Res..

[34] Sager R (2020). Auxin-dependent control of a plasmodesmal regulator creates a negative feedback loop modulating lateral root emergence. Nat. Commun..

[35] Crooks GE, Hon G, Chandonia JM, Brenner SE (2004). WebLogo: a sequence logo generator. Genome Res..

[36] Finn RD, Clements J, Eddy SR (2011). HMMER web server: interactive sequence similarity searching. Nucleic Acids Res..

