# Supplementary material for: An evolutionarily conserved motif is required for Plasmodesmata-located protein 5 to regulate cell-to-cell movement
Source: Commun Biol. 2020 Jun 5;3:291. doi: 10.1038/s42003-020-1007-0 (PMC7275062; doi:10.1038/s42003-020-1007-0)
Supplement: Supplementary file 1 — Supplementary Information [file 42003_2020_1007_MOESM1_ESM.pdf]

Supplementary Figures

Supplementary Figure 1

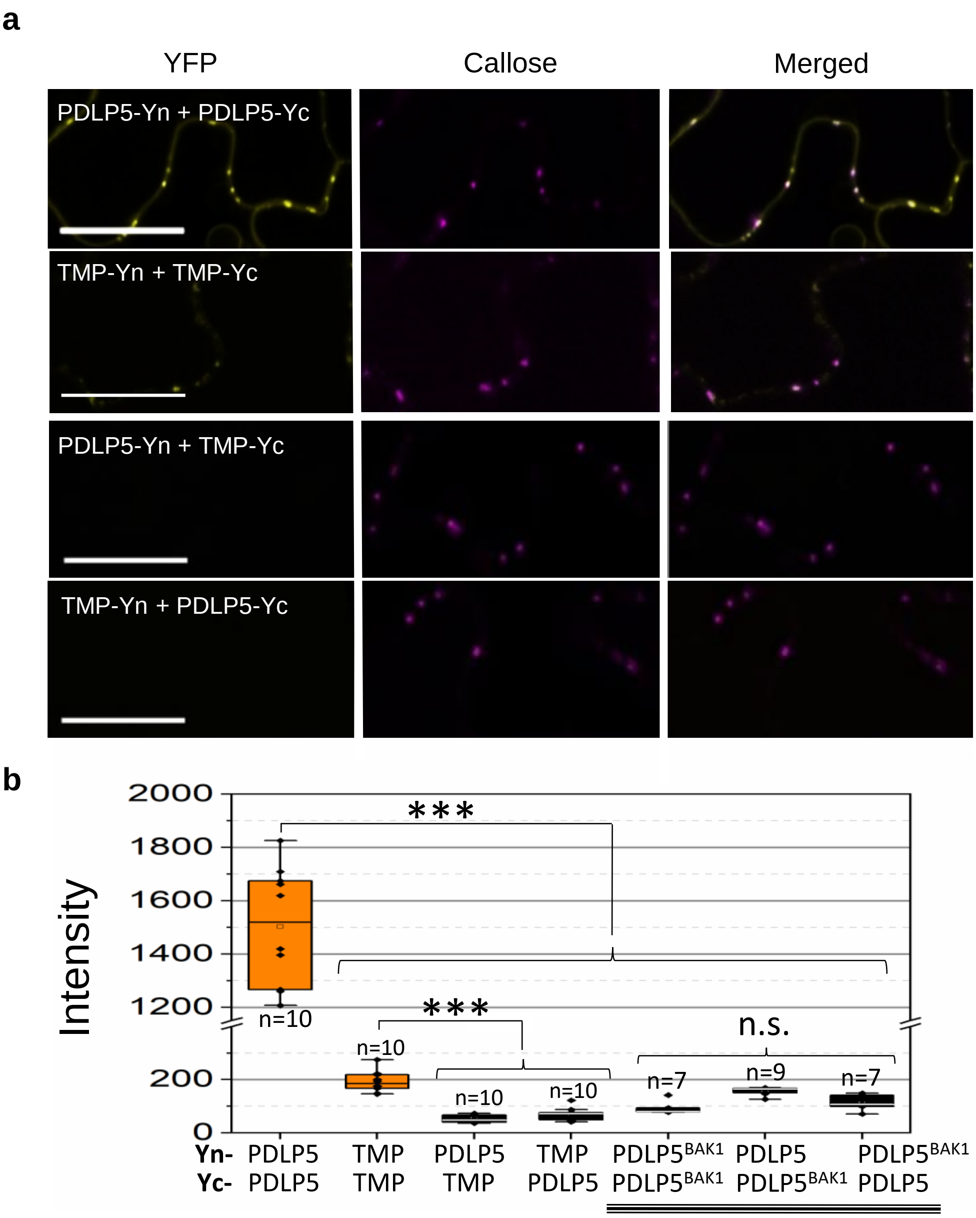

a. Confocal images showing positive or negative YFP signals from specific BiFC pairs and aniline blue-stained callose depositions (false-colored in magenta). Strong punctate YFP signals were detected only from the PDLP5-Yn/PDLP5-Yc pair, which co-localize at plasmodesmata labeled with callose. The other BiFC pairs produce extremely low or undetectable YFP signals. Size bars, 20 µm. **b.** Graph showing fluorescent intensity. n, the number of YFP puncta at plasmodesmata analyzed. Plasmodesmata were identified using aniline blue staining and randomly chosen from 3-5 single scan confocal images for fluorescence intensity quantification purpose. Regions of interest over plasmodesmata were manually drawn to calculate the average YFP intensity using ImageJ. Triple lines indicate the BiFC pairs, representative confocal images of which are presented in Supplemental figure 5. Statistical analysis was performed using one-way ANOVA, followed by pairwise Tukey's test at the probability of $p < 0.001$. Asterisks, significant difference; n.s., no significant difference; Error bars, standard deviation.

Supplementary Figure 2

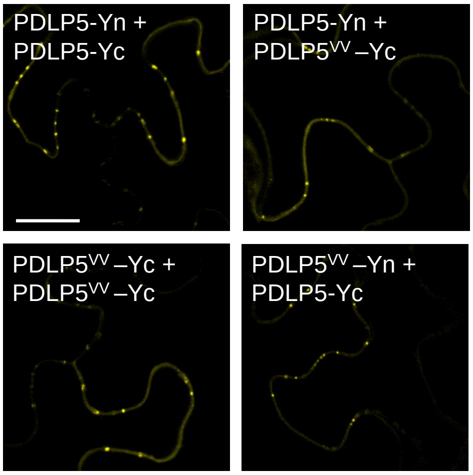

Confocal images showing strong punctate YFP signals at plasmodesmata from BiFC pairs of PDLP5 and PDLP5^{VV} homomers and heteromers. Size bar, 20 µm, common to all panels.

Wang & Robles-Luna et al., Comms. Biol.2020

Supplementary Figure 3

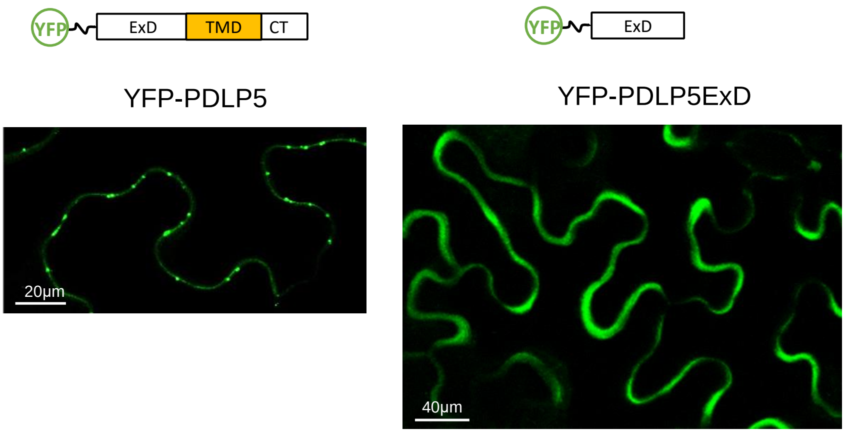

Confocal images showing PD and cell wall localization of YFP-PDLP5 and YFP-PDLP5ExD, respectively, in tobacco epidermal cells. The YFP version used is Citrine.

Supplementary Figure 4

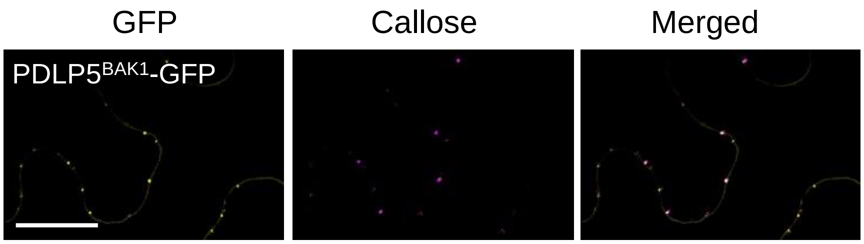

Co-localization of PDLP5^{BAK1}-GFP (false-colored in yellow) with callose (false-colored in magenta) at plasmodesmata. Size bar, 20 µm, common to all panels.

Supplementary Figure 5

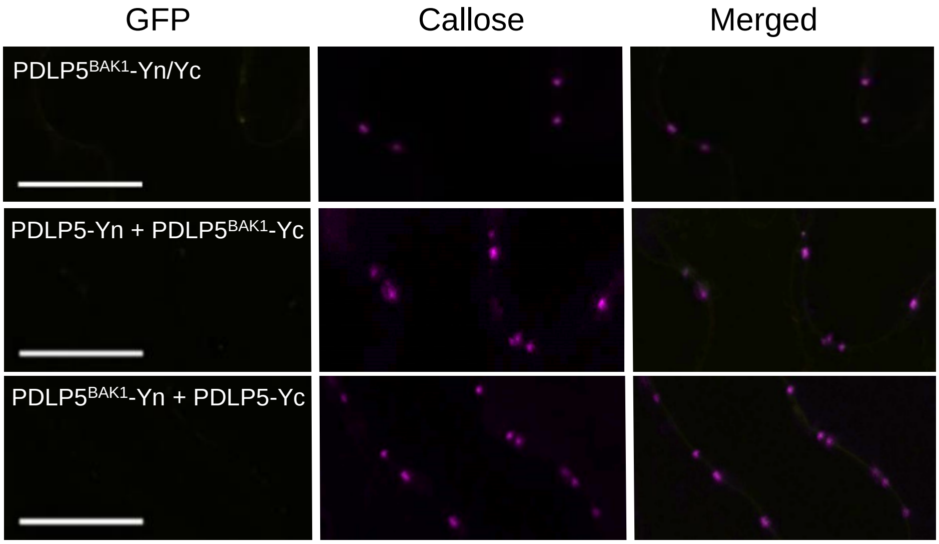

Confocal images showing no detectable YFP fluorescence from BiFC pairs examined. Callose staining reveals plasmodesmata between epidermal cells (false-colored in magenta). Size bars, 20 µm.

Supplementary Figure 6

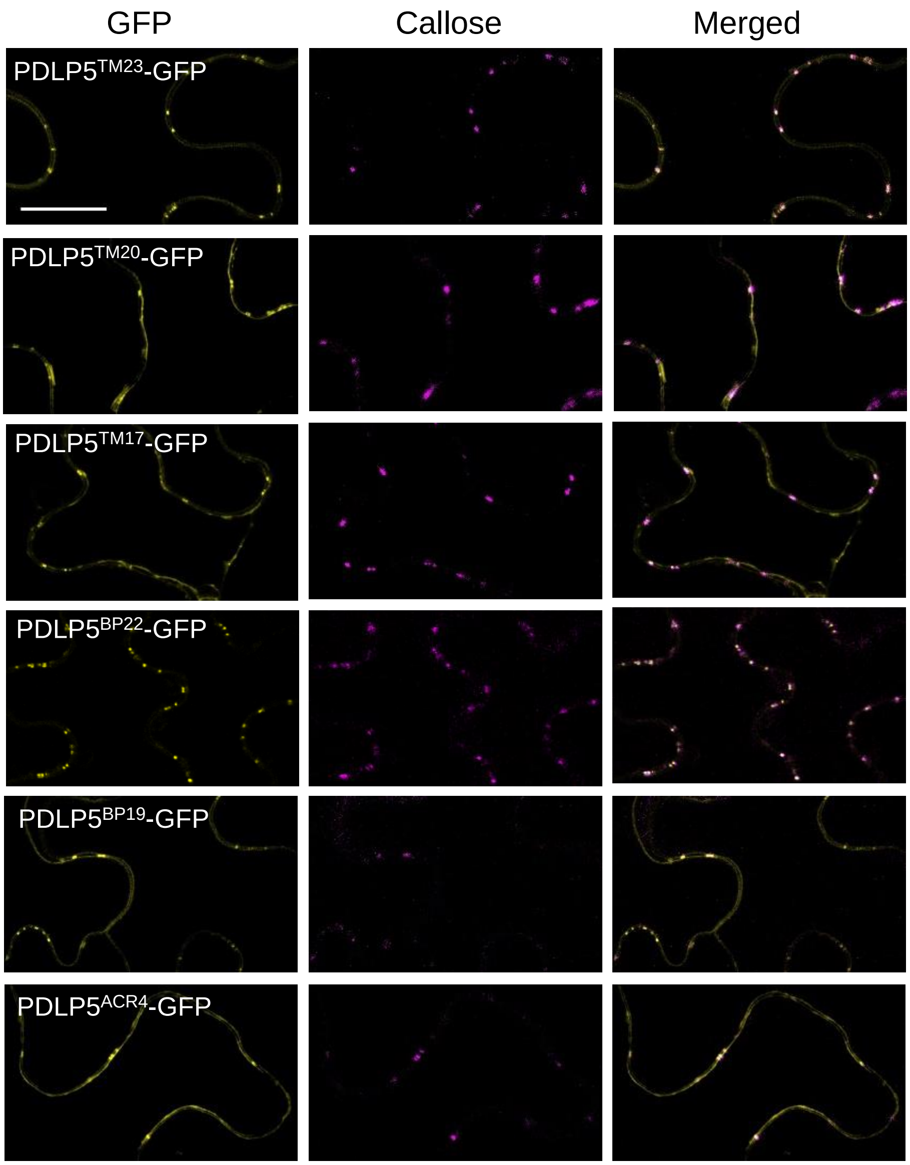

Confocal images showing co-localization of PDLP5 TMD swap mutants (false-colored in yellow) with callose depositions at plasmodesmata (false-colored in magenta). Size bar, 20 µm, common to all panels.

Supplementary Figure 7

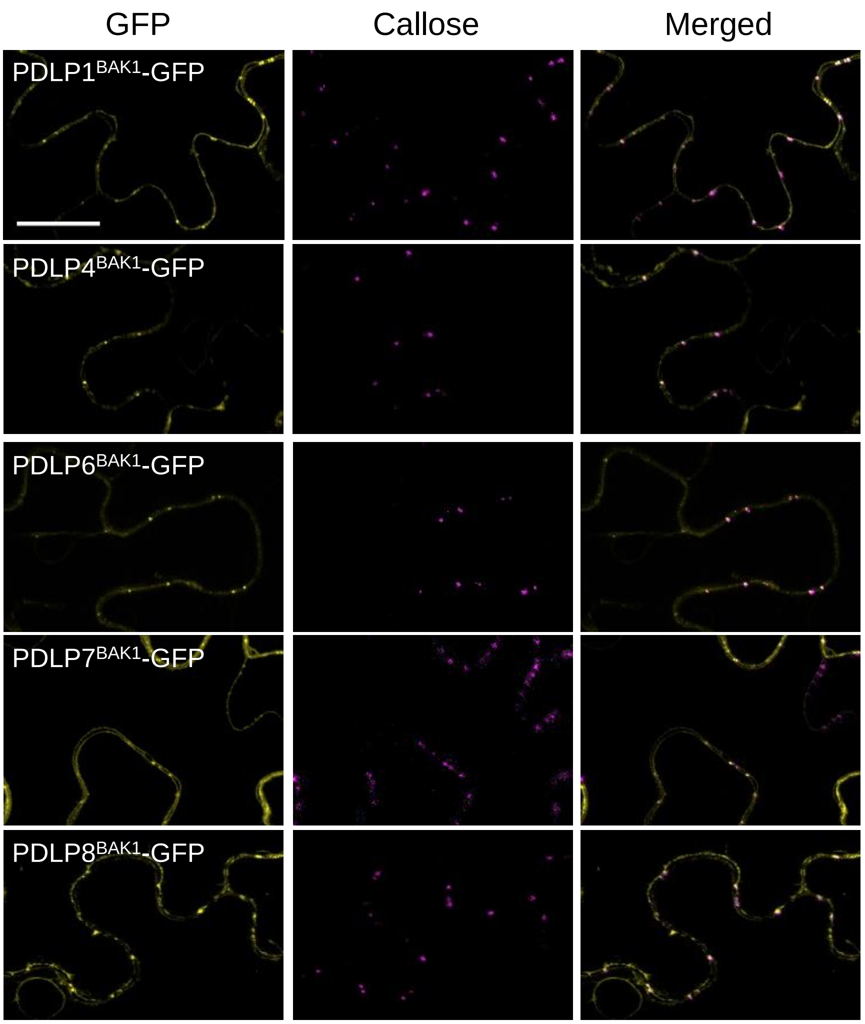

Confocal images showing co-localization of TMD swap mutants derived from PDLP1, 4, 6, 7, and 8 (false-colored in yellow) with callose depositions (false-colored in magenta) at plasmodesmata. Size bar, 20 µm, common to all panels.

Supplementary Figure 8

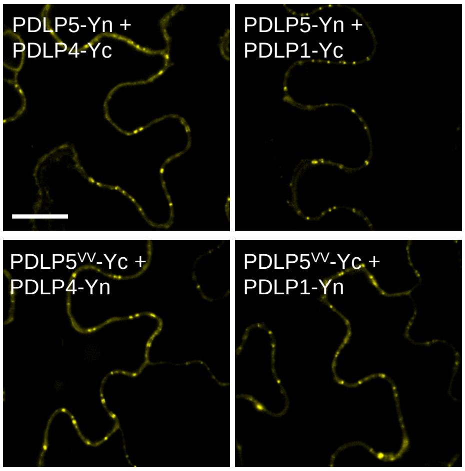

Confocal images showing positive BiFC signals at plasmodesmata in support of heteromeric interactions. Size bar, 20 µm, common to all panels.

Supplementary Figure 9

PDLP5^{ACR4} –Yn +
PDLP5^{ACR4} –Yc

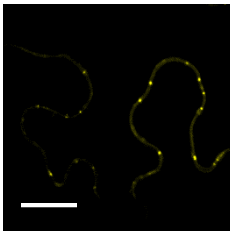

Confocal images showing positive BiFC signals at plasmodesmata, which support homomeric interactions of PDLP5^{ACR4}. Size bar, 20 µm.

Supplementary Notes, Discussion, Methods

TMD Sequence alignment

```
Reference sequence (1): sp|Q8GXV7|PDLP1_ARATH
Identities normalised by aligned length.
Colored by: identity

    consensus/100%                                               .shhhu.........h.....
    consensus/90%                                                hAlhlGhhsulshhllhh.hh
    consensus/80%                                                lAIllGhhAuluhlllhl.hh
    consensus/70%                                                lAIllGhhAuluhlllhL.Fh

                                          cov    pid   1 [         .         .] 21
  1 sp|Q8GXV7|PDLP1_ARATH            100.0% 100.0%    IALAVGGVFVLGFVIVCLLVL
  2 sp|Q6NM73|PDLP2_ARATH            100.0%  42.9%    VAIIVGGAAGVGFLVICLLFA
  3 sp|O22784|PDLP3_ARATH            100.0%  42.9%    VAIIVGGTAGVGFLVICLLFV
  4 sp|Q6E263|PDLP4_ARATH            100.0%  42.9%    LAIVVGGVAALVFVAIFFMFL
  5 sp|Q8GUJ2|PDLP5_ARATH            100.0%  23.8%    LAIIIGIVTLIILLVVFLAFV
  6 sp|Q9ZU94|PDLP6_ARATH            100.0%  23.8%    LAIIVGLIAGVTLLVVFLSFM
  7 sp|Q0WPN8|PDLP7_ARATH            100.0%  28.6%    FAIIIGLLAAVVLLIIFLLFL
  8 sp|Q6NKQ9|PDLP8_ARATH            100.0%  28.6%    IAIIVGVIAGFAILVVLLSLC
  9 tr|D7L246|D7L246_ARALL           100.0%  42.9%    LAIVVGGVAALVFVAIFFMFL
 10 tr|R0G8D6|R0G8D6_9BRAS           100.0%  38.1%    LAIVVGGVAALVLVAIFFMFL
 11 tr|A0A078IH97|A0A078IH97_BRANA 100.0%  47.6%    LAIVVGGVAALVFVAIFFLFL
 12 tr|A0A0D3CMU2|A0A0D3CMU2_BRAOL 100.0%  47.6%    LAIVVGGVAALVFVAIFFLFL
 13 tr|V4M0M8|V4M0M8_EUTSA           100.0%  42.9%    LAIVVGGVAALVFVAIFFMFL
 14 tr|A0A1S4AU48|A0A1S4AU48_TOBAC 100.0%  23.8%    VAIVAGGIVATIFLGVVYYFM
 15 tr|A0A1U7X9A1|A0A1U7X9A1_NICSY 100.0%  23.8%    VAIVAGGIVATIFLGVVYYFM
 16 tr|A0A1J6IG11|A0A1J6IG11_NICAT 100.0%  14.3%    VAIVAGGIVATILLGIVYYFM
 17 tr|A0A1S3ZHH4|A0A1S3ZHH4_TOBAC 100.0%  19.0%    VAIVAGGIVATILLGVVYYFM
 18 tr|K4C2R8|K4C2R8_SOLLC           100.0%  19.0%    AAIVIGGIVATILLGVVYYFF
 19 tr|A0A022RGN3|A0A022RGN3_ERYGU 100.0%  23.8%    VAIVVGGLAVVSLGLALCYCT
 20 tr|A0A2U1P4D1|A0A2U1P4D1_ARTAN 100.0%  19.0%    AAMVVGVGVALALLTTLCFCI
 21 tr|A0A251UQR0|A0A251UQR0_HELAN 100.0%  14.3%    AAMVVGVGVAIALLSTLCYCI
 22 tr|A0A2J6LX91|A0A2J6LX91_LACSA 100.0%  19.0%    VAMVVGIGVALALLFALCYCI
 23 tr|A0A0A0LMT6|A0A0A0LMT6_CUCSA 100.0%  28.6%    VAIVLGGAATLGLAFILLIFF
 24 tr|A0A2P5C3B3|A0A2P5C3B3_PARAD 100.0%  33.3%    LAIVLGGGAALCFGFIFLLFI
 25 tr|A0A2P5FYP9|A0A2P5FYP9_TREOI 100.0%  28.6%    LAIVLGGAAALCFGFIFMLFI
 26 tr|W9RHY0|W9RHY0_9ROSA           100.0%  38.1%    VAIVLGGAAALVVGYIFLLVL
 27 tr|M5WQ65|M5WQ65_PRUPE           100.0%  38.1%    VAIVVGGAAALCLGFVFLLFV
 28 tr|A0A2P6R5G4|A0A2P6R5G4_ROSCH 100.0%  42.9%    LAIVVGGAAALFFGLIFLLFL
 29 tr|A0A0R0ERG8|A0A0R0ERG8_SOYBN 100.0%  33.3%    AAIIVGGAAALFLGFAFLSLL
 30 tr|K7LJU0|K7LJU0_SOYBN           100.0%  33.3%    AAIIVGGAATLFLGFAFLSLL
 31 tr|A0A0L9THI0|A0A0L9THI0_PHAAN 100.0%  23.8%    AAIIVGGAVAVFVGFALMSML
 32 tr|V7BGG4|V7BGG4_PHAVU           100.0%  23.8%    AAIIVGGAAAVFVGFALISML
 33 tr|G7I6T5|G7I6T5_MEDTR           100.0%  28.6%    IAIIVGGGAILFMGLIVVSLI
 34 tr|A0A1S2XWE0|A0A1S2XWE0_CICAR 100.0%  33.3%    IAIIVGGAAALLLGLIFVSLL
 35 tr|A0A2I4F5T6|A0A2I4F5T6_JUGRE 100.0%  33.3%    TAIVVGGAAAIFFGFIFLLCI
 36 tr|A0A0D2TMG3|A0A0D2TMG3_GOSRA 100.0%  28.6%    VAIVLGGAAALFIGYILFLSL
 37 tr|A0A1U8LLU8|A0A1U8LLU8_GOSHI 100.0%  28.6%    VAIVLGGAAALFIGYILFLSL
 38 tr|A0A1U8J9W2|A0A1U8J9W2_GOSHI 100.0%  28.6%    VAIVLGGAAALFIGYILFLSL
 39 tr|A0A061F8L4|A0A061F8L4_THECC 100.0%  33.3%    LAIVLGGAAALFIGYIFLLSL
 40 tr|A0A1Q3CRY1|A0A1Q3CRY1_CEPFO 100.0%  28.6%    EAIVLGGAASLVFGFIFLKFI
 41 tr|A0A2C9VG76|A0A2C9VG76_MANES 100.0%  19.0%    VAIVLGAAAAALSGGFIFLKL
 42 tr|A0A2C9VG63|A0A2C9VG63_MANES 100.0%  19.0%    VAIVLGAAAAALSGGFIFLKL
 43 tr|A0A067JW46|A0A067JW46_JATCU 100.0%  23.8%    VAMVLGGAVALLVGFILLKFK
 44 tr|A0A2K1Y135|A0A2K1Y135_POPTR 100.0%  38.1%    VAIVVGGAVVLGVGFILLKFF
 45 tr|A0A2H5NT33|A0A2H5NT33_CITUN 100.0%  33.3%    VAIVLGGVAALALGFIFLSFL
 46 tr|V4TGJ8|V4TGJ8_9ROSI           100.0%  33.3%    VAIVLGGVAALALGFIFLSFL
 47 tr|A0A059B654|A0A059B654_EUCGR 100.0%  33.3%    IAIVLGGAGALVLGFLFLLLI
 48 tr|D7KDF7|D7KDF7_ARALL           100.0%  42.9%    VAIIVGGAAGVGFLVICLLFA
 49 tr|R0GQX0|R0GQX0_9BRAS           100.0%  42.9%    VAIIVGGAAGVGFLVICLLFA
 50 tr|A0A078FUW4|A0A078FUW4_BRANA 100.0%  42.9%    VAIIVGGAAGVGFLVICLLFA
 51 tr|M4DFQ0|M4DFQ0_BRARP           100.0%  42.9%    VAIIVGGAAGVGFLVICLLFA
 52 tr|A0A0D3C7J6|A0A0D3C7J6_BRAOL 100.0%  42.9%    VAIIVGGAAGVGFLVICLLFA
 53 tr|V4KYM7|V4KYM7_EUTSA           100.0%  42.9%    VAIIVGGAAGVGFLVICLLFA
 54 tr|D7LFS7|D7LFS7_ARALL           100.0%  42.9%    VAIIVGGTAGVGFLVICLLFV
 55 tr|R0HDH0|R0HDH0_9BRAS           100.0%  42.9%    VAIIVGGTAGVGFLVICLLFV
 56 tr|M4CMR7|M4CMR7_BRARP           100.0%  52.4%    VAIIVGGTAGLGFLIICLLFV
 57 tr|A0A078FVM9|A0A078FVM9_BRANA 100.0%  52.4%    VAIIVGGTAGLGFLIICLLFV
```

58	tr|A0A078G475|A0A078G475_BRANA	100.0%	52.4%	VAIIVGGTAGLGFLIICLLFV
59	tr|A0A0D3BSG4|A0A0D3BSG4_BRAOL	100.0%	52.4%	VAIIVGGTAGLGFLIICLLFV
60	tr|V4M6L1|V4M6L1_EUTSA	100.0%	42.9%	VAIIVGGTAGVGFLVICLLFV
61	tr|A0A2H5P2Y4|A0A2H5P2Y4_CITUN	100.0%	42.9%	VAIILGGAAGVGFVVICLLFA
62	tr|V4U2V7|V4U2V7_9ROSI	100.0%	42.9%	VAIILGGAAGVGFVVICLLFA
63	tr|A0A067DXI7|A0A067DXI7_CITSI	100.0%	42.9%	VAIILGGAAGVGFVVICLLFA
64	tr|B9SQT8|B9SQT8_RICCO	100.0%	38.1%	VAIILGGAAGVGFLVICLLFA
65	tr|A0A2C9UET4|A0A2C9UET4_MANES	100.0%	38.1%	VAIVLGGAAAVGFLAICLLFA
66	tr|A0A2C9W7W5|A0A2C9W7W5_MANES	100.0%	38.1%	VAIILGGAAGVGFLVICLLFA
67	tr|A0A2C9W9D8|A0A2C9W9D8_MANES	100.0%	38.1%	VAIILGGAAGVGFLVICLLFA
68	tr|A0A067KM14|A0A067KM14_JATCU	100.0%	38.1%	VAIILGGAVGVGFVVICLMFA
69	tr|A0A2K1YPY8|A0A2K1YPY8_POPTR	100.0%	38.1%	VAIIVGGAAGVGFLVIFLLFA
70	tr|A0A1Q3C256|A0A1Q3C256_CEPFO	100.0%	33.3%	VAIILGGAAGVGFLVICLMFA
71	tr|A0A0A0LLG0|A0A0A0LLG0_CUCSA	100.0%	38.1%	VAVILGGAAGVGFLVICLLFI
72	tr|A0A1S3CD12|A0A1S3CD12_CUCME	100.0%	38.1%	VAVILGGAAGVGFLVICLLFI
73	tr|A0A059ATQ2|A0A059ATQ2_EUCGR	100.0%	38.1%	VAIILGGAAGVGFLVICLLFA
74	tr|A0A059AUI3|A0A059AUI3_EUCGR	100.0%	38.1%	VAIILGGAAGVGFLVICLLFA
75	tr|G7I3U3|G7I3U3_MEDTR	100.0%	33.3%	VAIILGGIAAVAFLVICLLFA
76	tr|A0A1S3E2X1|A0A1S3E2X1_CICAR	100.0%	33.3%	VAIILGGIAGVAFLVICLLFA
77	tr|I1LCF3|I1LCF3_SOYBN	100.0%	28.6%	VAIILGGAAGVAFLVICLMFA
78	tr|I1NHZ5|I1NHZ5_SOYBN	100.0%	33.3%	VAIIVGGAAGVAFLVICLMFA
79	tr|A0A151T156|A0A151T156_CAJCA	100.0%	28.6%	VAIILGGLAGVAFLVICLMFA
80	tr|A0A0L9VBS0|A0A0L9VBS0_PHAAN	100.0%	38.1%	VAIILGGAAGVAFVVICLLFA
81	tr|A0A1S3V308|A0A1S3V308_VIGRR	100.0%	38.1%	VAIILGGAAGVAFVVICLLFA
82	tr|V7BGV8|V7BGV8_PHAVU	100.0%	38.1%	VAIILGGAAGVAFVVICLLFA
83	tr|I1LAQ4|I1LAQ4_SOYBN	100.0%	28.6%	AAIILGGAAAVAFLVILLLFA
84	tr|K7LJ71|K7LJ71_SOYBN	100.0%	28.6%	AAIILGGAAAVAFLVILLLFA
85	tr|I1NEM6|I1NEM6_SOYBN	100.0%	28.6%	AAIILGGAVAVAFLVILLLFA
86	tr|K7N2B1|K7N2B1_SOYBN	100.0%	28.6%	AAIILGGAVAVAFLVILLLFA
87	tr|A0A1S3V1E4|A0A1S3V1E4_VIGRR	100.0%	47.6%	AAIIVGGVATVAFVVICLLFA
88	tr|A0A0L9TVD5|A0A0L9TVD5_PHAAN	100.0%	47.6%	AAIIVGGVAAVAFVVICLLFA
89	tr|A0A1S3V1B1|A0A1S3V1B1_VIGRR	100.0%	47.6%	AAIIVGGVATVAFVVICLLFA
90	tr|V7BHL9|V7BHL9_PHAVU	100.0%	38.1%	AAIILGGVAGVTFLVICLLFA
91	tr|A0A2I4DKT2|A0A2I4DKT2_JUGRE	100.0%	38.1%	VAIILGGAAGVGFLVICLLFA
92	tr|A0A2I4EL37|A0A2I4EL37_JUGRE	100.0%	38.1%	VAIILGGAAGVGFLLICLLFA
93	tr|A0A2P6QRH9|A0A2P6QRH9_ROSCH	100.0%	33.3%	VAIILGGAAGVGFLVIFLLFA
94	tr|A0A251QSV4|A0A251QSV4_PRUPE	100.0%	33.3%	VAIILGGAAGVGFLVIFLLFA
95	tr|A0A2P5ANR5|A0A2P5ANR5_PARAD	100.0%	38.1%	VAIILGGAAGVGFLVICLLFA
96	tr|A0A2P5FAP0|A0A2P5FAP0_TREOI	100.0%	38.1%	VAIILGGAAGVGFLVICLLFA
97	tr|A0A1S3AW38|A0A1S3AW38_CUCME	100.0%	33.3%	VAIILGATAGVAFVVICLLFA
98	tr|A0A1U8LW28|A0A1U8LW28_GOSHI	100.0%	23.8%	VAIILGGAAGIGFIVILMMFA
99	tr|A0A1U8LSA6|A0A1U8LSA6_GOSHI	100.0%	23.8%	VAIILGGAAGIGFIVILMMFA
100	tr|A0A0D2S9A1|A0A0D2S9A1_GOSRA	100.0%	23.8%	VAIILGGAAGIGFIVILMMFA
101	tr|A0A1U8LMN5|A0A1U8LMN5_GOSHI	100.0%	19.0%	VAIILGAAAGIGFIVILMMFA
102	tr|A0A0D2T9L7|A0A0D2T9L7_GOSRA	100.0%	28.6%	VAIILGGAASVGFLVILLMFA
103	tr|A0A0D2UV82|A0A0D2UV82_GOSRA	100.0%	28.6%	VAIILGGAASVGFLVILLMFA
104	tr|A0A1U8IIG0|A0A1U8IIG0_GOSHI	100.0%	28.6%	VAIILGGAASVGFLVILLMFA
105	tr|A0A1U8IIH6|A0A1U8IIH6_GOSHI	100.0%	28.6%	VAIILGGAASVGFLVILLMFA
106	tr|A0A1U8KAN6|A0A1U8KAN6_GOSHI	100.0%	28.6%	VAIILGGAASVGFLVILLMFA
107	tr|A0A1U8KAN4|A0A1U8KAN4_GOSHI	100.0%	28.6%	VAIILGGAASVGFLVILLMFA
108	tr|A0A061EMX4|A0A061EMX4_THECC	100.0%	28.6%	VAIILGGAAGVGFLVILLMFA
109	tr|A0A061EPB1|A0A061EPB1_THECC	100.0%	28.6%	VAIILGGAAGVGFLVILLMFA
110	tr|A0A1R3IVG6|A0A1R3IVG6_COCAP	100.0%	33.3%	VAIIVGGAAGVGFLVILLMFA
111	tr|D7SZN7|D7SZN7_VITVI	100.0%	47.6%	VAIIVGGAAALGFVVICMLFA
112	tr|A0A2G2ZL38|A0A2G2ZL38_CAPAN	100.0%	42.9%	VAIIVGGVAGVGFILICVLFA
113	tr|A0A2G3CJD7|A0A2G3CJD7_CAPCH	100.0%	42.9%	VAIIVGGVAGVGFILICVLFA
114	tr|K4BDZ3|K4BDZ3_SOLLC	100.0%	38.1%	VAIILGGVAGVGFILICVLFA
115	tr|M1B1G2|M1B1G2_SOLTU	100.0%	33.3%	VAIILGGVAGVAFILICVLFA
116	tr|A0A1S3Y935|A0A1S3Y935_TOBAC	100.0%	47.6%	VAIIVGGVAAAGFLVICLLFA
117	tr|A0A1S3YA29|A0A1S3YA29_TOBAC	100.0%	47.6%	VAIIVGGVAAAGFLVICLLFA
118	tr|A0A1U7XRW3|A0A1U7XRW3_NICSY	100.0%	47.6%	VAIIVGGVAAAGFLVICLLFA
119	tr|A0A1J6ISX1|A0A1J6ISX1_NICAT	100.0%	47.6%	VAIIVGGVAAAGFLVICLLFA
120	tr|M1BFX9|M1BFX9_SOLTU	100.0%	33.3%	VAIILGGAAGVGFLVICMLFA
121	tr|M1BFX7|M1BFX7_SOLTU	100.0%	33.3%	VAIILGGAAGVGFLVICMLFA
122	tr|K4CW94|K4CW94_SOLLC	100.0%	33.3%	VAIILGGAAGVGFLVICMLFA
123	tr|A0A2G3CR65|A0A2G3CR65_CAPCH	100.0%	33.3%	VAIILGGAAGVGFLVICMLFA
124	tr|A0A1S3YE83|A0A1S3YE83_TOBAC	100.0%	38.1%	VAIILGGAAGVGFLVICLLFA
125	tr|A0A1U7Y7I1|A0A1U7Y7I1_NICSY	100.0%	38.1%	VAIILGGAAGVGFLVICLLFA
126	tr|A0A1S3XXN0|A0A1S3XXN0_TOBAC	100.0%	38.1%	VAIILGGAAGVGFLVICLLFA
127	tr|A0A314LBP3|A0A314LBP3_NICAT	100.0%	38.1%	VAIILGGAAGVGFLVICLLFA
128	tr|A0A175YPQ9|A0A175YPQ9_DAUCA	100.0%	33.3%	VAIILGGAAGVGFLVICMLFA

129	tr|A0A164T7E8|A0A164T7E8_DAUCA	100.0%	38.1%	VAIILGGAAGVGFLIICMLFA
130	tr|A0A022R1A6|A0A022R1A6_ERYGU	100.0%	38.1%	VAIIVGGAAGVGFLVIFLLFA
131	tr|A0A022QXH5|A0A022QXH5_ERYGU	100.0%	38.1%	VAIIVGGAAGVGFLVIFLLFA
132	tr|A0A022RVE8|A0A022RVE8_ERYGU	100.0%	38.1%	VAIILGGAAGVGFLVICLLFA
133	tr|A0A2U1QL79|A0A2U1QL79_ARTAN	100.0%	33.3%	VAIILGGAAGVGFIVICILFV
134	tr|A0A251SFN9|A0A251SFN9_HELAN	100.0%	28.6%	VAIILGGAAGVGFLVIFVLFA
135	tr|A0A103YFC6|A0A103YFC6_CYNCS	100.0%	33.3%	VAIILGGAAGVGFIVICILFA
136	tr|A0A2J6KFB4|A0A2J6KFB4_LACSA	100.0%	38.1%	VAIFLGGAAGVGFLVICLLFA
137	tr|A0A2U1QLV7|A0A2U1QLV7_ARTAN	100.0%	42.9%	VAIILGGAAGVGFLIVFLLIA
138	tr|A0A2J6MGQ8|A0A2J6MGQ8_LACSA	100.0%	42.9%	AAIILGGAAGVGFLIVLLLIA
139	tr|A0A251VKT4|A0A251VKT4_HELAN	100.0%	42.9%	VAIILGGAAGVGFIIVILLIA
140	tr|A0A0K9QH36|A0A0K9QH36_SPIOL	100.0%	28.6%	VAIIVGVTAGVAFLIIMVLFI
141	tr|A0A1U8APP6|A0A1U8APP6_NELNU	100.0%	47.6%	IAIVVGAAAAVGFVIICLMFI
142	tr|A0A200PVS1|A0A200PVS1_9MAGN	100.0%	42.9%	IAIVVGGAAGVGFGVICLMFI
143	tr|A0A2G5CYA1|A0A2G5CYA1_AQUCA	100.0%	52.4%	IAIVVGGVAAVGFAFICLMCL
144	tr|M0SY29|M0SY29_MUSAM	100.0%	47.6%	VAIVVGGAAGVGFFIICLLFA
145	tr|M0RW07|M0RW07_MUSAM	100.0%	47.6%	VAIVVGGAAGVGFLIICLLFA
146	tr|M0RNT0|M0RNT0_MUSAM	100.0%	42.9%	VAIVVGGAAGVGFLVICLLFA
147	tr|M0TPW7|M0TPW7_MUSAM	100.0%	42.9%	VAIVVGGAAGVGFLVICLLFA
148	tr|A0A2H3XKV6|A0A2H3XKV6_PHODC	100.0%	47.6%	VAIVVGGAAALGFLVICLLFA
149	tr|B8AMV6|B8AMV6_ORYSI	100.0%	47.6%	VAIVLGGAVGLGFVVICLLFA
150	tr|A0A0D3FDM0|A0A0D3FDM0_9ORYZ	100.0%	47.6%	VAIVLGGAVGLGFVVICLLFA
151	tr|I1P7F1|I1P7F1_ORYGL	100.0%	47.6%	VAIVLGGAVGLGFVVICLLFA
152	tr|Q0DVA3|Q0DVA3_ORYSJ	100.0%	47.6%	VAIVLGGAVGLGFVVICLLFA
153	tr|A0A0E0K8L7|A0A0E0K8L7_ORYPU	100.0%	47.6%	VAIVLGGAVGLGFVVICLLFA
154	tr|A0A0D9VPD4|A0A0D9VPD4_9ORYZ	100.0%	47.6%	VAIVLGGAVGLGFVVICLLFA
155	tr|A0A096QYG3|A0A096QYG3_MAIZE	100.0%	47.6%	VAIVLGGAVGLGFVVICLLFA
156	tr|C5WYW6|C5WYW6_SORBI	100.0%	47.6%	VAIVLGGAVGLGFVVICLLFA
157	tr|A0A2T7CHT7|A0A2T7CHT7_9POAL	100.0%	38.1%	VAIVLGGAVGVGFLVICLLFA
158	tr|A0A3B6JPB3|A0A3B6JPB3_WHEAT	100.0%	42.9%	VAIVLGGALALGFLVICLLFA
159	tr|A0A3B6KTM6|A0A3B6KTM6_WHEAT	100.0%	42.9%	VAIVLGGALALGFLVICLLFA
160	tr|A0A3B6IWS5|A0A3B6IWS5_WHEAT	100.0%	47.6%	VAIVLGGALVLGFLVICLLFA
161	tr|I1H9X2|I1H9X2_BRADI	100.0%	42.9%	VAIVLGGALALGFLVICLLFA
162	tr|D7MN47|D7MN47_ARALL	100.0%	100.0%	IALAVGGVFVLGFVIVCLLVL
163	tr|A0A078G755|A0A078G755_BRANA	100.0%	95.2%	IALAVGGVAVLGFVIVCLLVL
164	tr|M4EY19|M4EY19_BRARP	100.0%	95.2%	IALAVGGVAVLGFVIVCLLVL
165	tr|A0A0D3D8S3|A0A0D3D8S3_BRAOL	100.0%	95.2%	IALAVGGVAVLGFVIVCLLVL
166	tr|V4LXJ0|V4LXJ0_EUTSA	100.0%	90.5%	IALVVGGVAVLGFVIVCLLVL
167	tr|A0A059A1T0|A0A059A1T0_EUCGR	100.0%	57.1%	VAVVVGGVAAAGFGIVCLLFA
168	tr|A0A067K2H9|A0A067K2H9_JATCU	100.0%	61.9%	VAIAVGGVAALGFLIVCLMFV
169	tr|B9SKN1|B9SKN1_RICCO	100.0%	61.9%	VAIAVGGVAALGFLVVCLLFA
170	tr|A0A2C9UPT8|A0A2C9UPT8_MANES	100.0%	47.6%	VAIVVGGIAAFGFLIVCLMFI
171	tr|A0A2K2BPQ2|A0A2K2BPQ2_POPTR	100.0%	61.9%	VAIAVGGVAALGFGVVCLLFV
172	tr|A0A1Q3CSL9|A0A1Q3CSL9_CEPFO	100.0%	52.4%	VAIAVGGVAALGLLIVILMFI
173	tr|A0A1U8LG93|A0A1U8LG93_GOSHI	100.0%	66.7%	VAIAVGGVAALGFIIVCLMFL
174	tr|A0A1U8LC57|A0A1U8LC57_GOSHI	100.0%	66.7%	VAIAVGGVAALGFIIVCLMFL
175	tr|A0A1U8LC50|A0A1U8LC50_GOSHI	100.0%	66.7%	VAIAVGGVAALGFIIVCLMFL
176	tr|A0A0D2QV49|A0A0D2QV49_GOSRA	100.0%	66.7%	VAIAVGGVAALGFIIVCLMFL
177	tr|A0A1U8P645|A0A1U8P645_GOSHI	100.0%	66.7%	VAIAVGGVAALGFIIVCLMFL
178	tr|A0A1U8P636|A0A1U8P636_GOSHI	100.0%	66.7%	VAIAVGGVAALGFIIVCLMFL
179	tr|A0A061DWE8|A0A061DWE8_THECC	100.0%	66.7%	VAIAVGGVAALGFVVVCLMFL
180	tr|A0A061DNE9|A0A061DNE9_THECC	100.0%	66.7%	VAIAVGGVAALGFVVVCLMFL
181	tr|A0A0D2QS54|A0A0D2QS54_GOSRA	100.0%	61.9%	VAIAVGGVAGLGFLVVCLMFL
182	tr|A0A1U8NZH4|A0A1U8NZH4_GOSHI	100.0%	61.9%	VAIAVGGVAGLGFLVVCLMFL
183	tr|A0A1R3H3X6|A0A1R3H3X6_COCAP	100.0%	61.9%	VAIAVGGVAALGFIVVCLMFL
184	tr|A0A2P5B6F5|A0A2P5B6F5_PARAD	100.0%	61.9%	VAIAVGGIAALGFLIVCLLFV
185	tr|A0A2P5D0J4|A0A2P5D0J4_TREOI	100.0%	66.7%	VAIAVGGVAALGFLIVCLLFV
186	tr|W9QPA7|W9QPA7_9ROSA	100.0%	61.9%	VAIAVGGIAALGFLIVCLLFV
187	tr|A0A2I4DL71|A0A2I4DL71_JUGRE	100.0%	61.9%	VALAVGGLAAFGFVIVCFLFA
188	tr|A0A2I4F644|A0A2I4F644_JUGRE	100.0%	57.1%	VALAVGGLAAFGFIIVCFLFV
189	tr|A0A1S2XSF4|A0A1S2XSF4_CICAR	100.0%	66.7%	VALAVGGVAAIGFLIVCLMFL
190	tr|G7KXF5|G7KXF5_MEDTR	100.0%	66.7%	VALAVGGVAAFGFLIVCLMFL
191	tr|I1JP70|I1JP70_SOYBN	100.0%	76.2%	VALAVGGVAALGFLIVCLLFL
192	tr|I1N9U4|I1N9U4_SOYBN	100.0%	76.2%	VALAVGGVAALGFLIVCLLFL
193	tr|A0A1S3TNZ8|A0A1S3TNZ8_VIGRR	100.0%	71.4%	VALAVGGVAALGFLIVCMLFL
194	tr|A0A1S3TP43|A0A1S3TP43_VIGRR	100.0%	71.4%	VALAVGGVAALGFLIVCMLFL
195	tr|A0A0L9TI70|A0A0L9TI70_PHAAN	100.0%	71.4%	VALAVGGVAALGFLIVCMLFL
196	tr|V7CYX0|V7CYX0_PHAVU	100.0%	66.7%	VALAVGGVAALGFLIACMLCL
197	tr|I1LYP1|I1LYP1_SOYBN	100.0%	66.7%	VALAVGAFAALGFLIVCLLFL
198	tr|A0A151U4H1|A0A151U4H1_CAJCA	100.0%	66.7%	IALAVGAFAALGFLIACLLFL
199	tr|V7BJU6|V7BJU6_PHAVU	100.0%	66.7%	VALAVGGFAALGFLIACLLFL

```
200 tr|A0A1S3UZX1|A0A1S3UZX1_VIGRR 100.0%  52.4%    VALVVGGFAALGFLIASFLFL
201 tr|A0A151U4A1|A0A151U4A1_CAJCA 100.0%  52.4%    VALTVGGFGALALLIVCYLVI
202 tr|A0A2P6PUW5|A0A2P6PUW5_ROSCH 100.0%  52.4%    VAIVVGGVAAVGFVFVCLMFI
203 tr|A0A251N1H0|A0A251N1H0_PRUPE 100.0%  47.6%    VAIAVGGLAAFGFLVVCLMFV
204 tr|D7U362|D7U362_VITVI         100.0%  57.1%    VALVVGGVAALGFGIACLMFV
205 tr|A0A2H5NJ90|A0A2H5NJ90_CITUN 100.0%  52.4%    VAMAVGIVAAVGFVLVLLLFV
206 tr|V4VUP5|V4VUP5_9ROSI         100.0%  52.4%    VAMAVGIVAAVGFVLVLLLFV
207 tr|A0A1S3BH96|A0A1S3BH96_CUCME 100.0%  61.9%    VALAVGGFAALAFLIVCLLFV
208 tr|A0A0K9RBC7|A0A0K9RBC7_SPIOL 100.0%  42.9%    VAIVLGGTAAAGFVAVILLFT
209 tr|A0A118K7J9|A0A118K7J9_CYNCS 100.0%  47.6%    VAIVFGGLAGLGLVVAFLLVL
210 tr|A0A2J6JQ52|A0A2J6JQ52_LACSA 100.0%  47.6%    VAIVFGGLAGLGLVVAFLLVL
211 tr|A0A251V0R9|A0A251V0R9_HELAN 100.0%  47.6%    VAIVFGGLAALGLVVAFLLVL
212 tr|A0A251S5F2|A0A251S5F2_HELAN 100.0%  47.6%    VALVIGGIAGLFLGIAFLLVL
213 tr|A0A251TF84|A0A251TF84_HELAN 100.0%  42.9%    VAIIIGGLAGLFLAIAFLLVL
214 tr|A0A2U1Q656|A0A2U1Q656_ARTAN 100.0%  33.3%    VAIVFGGLAVVLLLVAFLLVV
215 tr|A0A164Z3Q2|A0A164Z3Q2_DAUCA 100.0%  52.4%    VAIVVGGLVGSGLFIACLLVL
216 tr|K4CAN0|K4CAN0_SOLLC         100.0%  33.3%    VAIVLGGLVGVGLGVACLLFT
217 tr|M1BS36|M1BS36_SOLTU         100.0%  33.3%    VAIVLGGLVGVGLGVACLLFT
218 tr|A0A2G2ZEJ6|A0A2G2ZEJ6_CAPAN 100.0%  33.3%    VAIVLGGLVGVGLGVACLLFT
219 tr|A0A1S3ZNA5|A0A1S3ZNA5_TOBAC 100.0%  38.1%    VAIVLGGMVGVGLGVACLLVT
220 tr|A0A1U7XIF5|A0A1U7XIF5_NICSY 100.0%  38.1%    VAIVLGGMVGVGLGVACLLVT
221 tr|A0A1S3XG98|A0A1S3XG98_TOBAC 100.0%  38.1%    VAIVLGGMVGVGLGVACLLVT
222 tr|A0A1S3ZRB8|A0A1S3ZRB8_TOBAC 100.0%  38.1%    VAIVLGGLVGVGLVLACLLFT
223 tr|A0A314L375|A0A314L375_NICAT 100.0%  38.1%    VAIVLGGLVGVGLVLACLLFT
224 tr|A0A1S4AM49|A0A1S4AM49_TOBAC 100.0%  38.1%    VAIVLGGLVGVGLVLACLLFT
225 tr|A0A1U7WXZ3|A0A1U7WXZ3_NICSY 100.0%  38.1%    VAIVLGGLVGVGLVLACLLFT
226 tr|A0A1S4ALZ3|A0A1S4ALZ3_TOBAC 100.0%  38.1%    VAIVLGGLVGVGLVLACLLFT
227 tr|A0A1U7WZ95|A0A1U7WZ95_NICSY 100.0%  38.1%    VAIVLGGLVGVGLVLACLLFT
228 tr|A0A022PR55|A0A022PR55_ERYGU 100.0%  42.9%    VAIAMGGLVSVGLVMACLLFT
229 tr|A0A2R6PEH4|A0A2R6PEH4_ACTCH 100.0%  57.1%    VAIVVGGAAGLGLGIACLLVL
230 tr|A0A2R6PF06|A0A2R6PF06_ACTCH 100.0%  66.7%    AAIVVGGVAGLGFVIACFLVL
231 tr|A0A2P6PYG2|A0A2P6PYG2_ROSCH 100.0%  19.0%    VAIIVGSIIGLILLGAVVIYI
232 tr|D7KYD3|D7KYD3_ARALL         100.0%  28.6%    LAIIIGIVTLIILLVVFLAFL
233 tr|R0I5Y2|R0I5Y2_9BRAS_altinit 100.0%  23.8%    LAIIIGMVTLIILLVVFLAFV
234 tr|R0HUA4|R0HUA4_9BRAS_altinit 100.0%  23.8%    LAIIIGMVTLIILLVVFLAFV
235 tr|A0A078FF48|A0A078FF48_BRANA 100.0%  19.0%    LVIIIGIITLVILLVVLLAFL
236 tr|A0A0D3CZN0|A0A0D3CZN0_BRAOL 100.0%  19.0%    LVIIIGIITLVILLVVLLAFL
237 tr|M4DI67|M4DI67_BRARP         100.0%  14.3%    LVIIIGIITLVILLVLLLAFL
238 tr|A0A078F8Y7|A0A078F8Y7_BRANA 100.0%  23.8%    LAIIIGIITLVILLVVFLAFL
239 tr|A0A0D3APP8|A0A0D3APP8_BRAOL 100.0%  23.8%    LAIIIGIITLVILLVVFLAFL
240 tr|D7LLU5|D7LLU5_ARALL         100.0%  23.8%    LAIIVGLIAGVTLLVVFLSFM
241 tr|R0FQT5|R0FQT5_9BRAS         100.0%  23.8%    LAIIVGLIAGVTLLVVFLSFM
242 tr|V4K6F3|V4K6F3_EUTSA         100.0%  23.8%    LAIIVGLIAGVTLLVVFLSFM
243 tr|A0A078FX03|A0A078FX03_BRANA 100.0%  23.8%    LAIIVGLIAGVTLLVVLLSFM
244 tr|A0A078HEP9|A0A078HEP9_BRANA 100.0%  23.8%    LAIIVGLIAGVTLLVVLLSFM
245 tr|A0A2P5ABF4|A0A2P5ABF4_PARAD 100.0%  28.6%    LAILIGLIAGVALLIVFLSFL
246 tr|A0A2P5BG41|A0A2P5BG41_TREOI 100.0%  28.6%    LAILIGLIAGVALLIVFLSFL
247 tr|W9SDN0|W9SDN0_9ROSA         100.0%  28.6%    LAILIGLIAGVALLIVFLSFL
248 tr|A9PGZ2|A9PGZ2_POPTR         100.0%  33.3%    LAILVGLIAGVALLIVFLAFL
249 tr|A0A2K1ZGL7|A0A2K1ZGL7_POPTR 100.0%  33.3%    LAILVGLIAGVALLIVFLAFL
250 tr|A0A2K1YS22|A0A2K1YS22_POPTR 100.0%  23.8%    LAILIGLIAAVALLIVFLSFF
251 tr|A0A067KPM7|A0A067KPM7_JATCU 100.0%  28.6%    LAILIGLIAGVALLIVFLSFL
252 tr|A0A2C9UGM7|A0A2C9UGM7_MANES 100.0%  28.6%    LAILIGLIAGVALLIVFLSFL
253 tr|B9SJ12|B9SJ12_RICCO         100.0%  28.6%    LAILIGLIAGVALLIVFLSFL
254 tr|A0A2C9U744|A0A2C9U744_MANES 100.0%  23.8%    LAILIGLIAGVTLLFVFLSCL
255 tr|A0A0D2SHR3|A0A0D2SHR3_GOSRA 100.0%  23.8%    LAILIGLIAGIALIIVFVSAL
256 tr|A0A0D2RM73|A0A0D2RM73_GOSRA 100.0%  23.8%    LAILIGLIAGIALIIVFVSAL
257 tr|A0A1U8HHY5|A0A1U8HHY5_GOSHI 100.0%  23.8%    LAILIGLIAGIALIIVFVSAL
258 tr|A0A1U8HHW2|A0A1U8HHW2_GOSHI 100.0%  23.8%    LAILIGLIAGIALIIVFVSAL
259 tr|A0A1U8JRZ1|A0A1U8JRZ1_GOSHI 100.0%  23.8%    LAILIGLIAGIALIIVFVSAL
260 tr|A0A1U8JWA4|A0A1U8JWA4_GOSHI 100.0%  23.8%    LAILIGLIAGIALIIVFVSAL
261 tr|A0A1U8NH80|A0A1U8NH80_GOSHI 100.0%  23.8%    LAILIGLIAAVALIILFLSFL
262 tr|A0A0D2Q7Y6|A0A0D2Q7Y6_GOSRA 100.0%  23.8%    LAILIGLIAAVALIILFLSFL
263 tr|A0A1R3GRE8|A0A1R3GRE8_COCAP 100.0%  23.8%    LAILIGLIAGVALLIVFVSFL
264 tr|A0A067FMV7|A0A067FMV7_CITSI 100.0%  14.3%    LAILIGMIAGIALLVIFLSSI
265 tr|A0A2H5PVU0|A0A2H5PVU0_CITUN 100.0%  14.3%    LAILIGMIAGIALLVIFLSSI
266 tr|A0A1Q3BFW8|A0A1Q3BFW8_CEPFO 100.0%  19.0%    LAILVGLIAAVALLVVFISFF
267 tr|A0A0L9T9S2|A0A0L9T9S2_PHAAN 100.0%  28.6%    LAILIGLIAGVALIIVFLSFL
268 tr|A0A1S3UI55|A0A1S3UI55_VIGRR 100.0%  28.6%    LAILIGLIAGVALIIVFLSFL
269 tr|V7CQL7|V7CQL7_PHAVU         100.0%  28.6%    LAILIGLIAGVALIIVFLSFL
270 tr|A0A0R4J3V7|A0A0R4J3V7_SOYBN 100.0%  28.6%    LAILIGLIAGVALIIVFLSFL
```

271	tr|I1K5Q6|I1K5Q6_SOYBN	100.0%	28.6%	LAILIGLIAGVALIIVFLSFL
272	tr|A0A151T3D1|A0A151T3D1_CAJCA	100.0%	28.6%	LAILIGLIAGVALIIVFLSFL
273	tr|A0A1S2YEQ9|A0A1S2YEQ9_CICAR	100.0%	23.8%	LAILIGLIAGVALIIIFLSFL
274	tr|G7LAI6|G7LAI6_MEDTR	100.0%	33.3%	LAILIGLIAGVGLIIVFLSFL
275	tr|M5XG08|M5XG08_PRUPE	100.0%	19.0%	LAILIGVIAGVALLVVFLSYF
276	tr|A0A2P6R3T2|A0A2P6R3T2_ROSCH	100.0%	19.0%	LAILIGIIAAVALLVVFLSYF
277	tr|A0A2I4DH21|A0A2I4DH21_JUGRE	100.0%	28.6%	LAIIIGSIAAVGLLIIFLSFL
278	tr|A0A059BFQ9|A0A059BFQ9_EUCGR	100.0%	33.3%	LAILIGLIAGVTLVIVFLSFL
279	tr|A0A0A0KRX8|A0A0A0KRX8_CUCSA	100.0%	28.6%	LAVIIGLIAGIAFIVVFLAFL
280	tr|A0A1S3CML3|A0A1S3CML3_CUCME	100.0%	28.6%	LAVIIGLIAGIAFIVVFLAFL
281	tr|A0A1S3CSR6|A0A1S3CSR6_CUCME	100.0%	19.0%	LAIIIGLIAAVALLILFITYL
282	tr|A0A1S3CSR3|A0A1S3CSR3_CUCME	100.0%	19.0%	LAIIIGLIAAVALLILFITYL
283	tr|A0A0A0KUP4|A0A0A0KUP4_CUCSA	100.0%	19.0%	LAIIIGLIAAVALLILFITYL
284	tr|A0A059D768|A0A059D768_EUCGR	100.0%	19.0%	VVILIGVIAGVAVVIIFLSLT
285	tr|A0A166EFB4|A0A166EFB4_DAUCA	100.0%	33.3%	LAILIGLIAGVALVIVFLSCL
286	tr|A0A175YGF1|A0A175YGF1_DAUCA	85.7%	19.0%	LAILIAIIAIVALVII---CL
287	tr|A0A2U1KZ05|A0A2U1KZ05_ARTAN	100.0%	28.6%	LAIIIGIIAGVAVLIVFLSML
288	tr|A0A2U1Q6G0|A0A2U1Q6G0_ARTAN	100.0%	28.6%	LAIIIGIIAGVAVLIVFLSML
289	tr|A0A251RUA1|A0A251RUA1_HELAN	100.0%	28.6%	LAIIIGIIAGVAVMIVFLSCL
290	tr|A0A175YQN9|A0A175YQN9_DAUCA	100.0%	19.0%	LAILIGLITGVILIIILLSGF
291	tr|A0A1S3ZWZ3|A0A1S3ZWZ3_TOBAC	100.0%	28.6%	LAITIGLIAGVAVLIVFLSIL
292	tr|A0A1U7Y2V4|A0A1U7Y2V4_NICSY	100.0%	28.6%	LAITIGLIAGVAVLIVFLSIL
293	tr|A0A1S3XGX5|A0A1S3XGX5_TOBAC	100.0%	33.3%	LAITIGLIAGVAVLIVFLSVL
294	tr|A0A314KV99|A0A314KV99_NICAT	100.0%	33.3%	LAITIGLIAGVAVLIVFLSVL
295	tr|A0A2G3CFE1|A0A2G3CFE1_CAPCH	100.0%	33.3%	LAITIGLIAGVAVLIVFLSVL
296	tr|M1CVR8|M1CVR8_SOLTU	100.0%	33.3%	LAITIGLIAGVAVLIVFLSVL
297	tr|M1CVR7|M1CVR7_SOLTU	100.0%	33.3%	LAITIGLIAGVAVLIVFLSVL
298	tr|K4BNV1|K4BNV1_SOLLC	100.0%	33.3%	LAITIGLIAGVALLIVFLSVL
299	tr|A0A022QQ91|A0A022QQ91_ERYGU	100.0%	28.6%	LAIFIGLVAAVAILIVFLSFF
300	tr|A0A2U1NS06|A0A2U1NS06_ARTAN	100.0%	28.6%	LAITLGVITGIILLIVCLSSI
301	tr|A0A2U1NRY8|A0A2U1NRY8_ARTAN	100.0%	28.6%	LAITLGVITGIILLIVCLSSI
302	tr|A0A251T132|A0A251T132_HELAN	100.0%	33.3%	LALTIGVITGVILLIVFLSSL
303	tr|A0A2J6KD26|A0A2J6KD26_LACSA	100.0%	23.8%	LAITIGVITGVILLIIFLSSL
304	tr|A0A2U1L579|A0A2U1L579_ARTAN	100.0%	19.0%	LAITIGSSVVGVVSSVGIVAA
305	tr|A0A2J6KD40|A0A2J6KD40_LACSA	100.0%	14.3%	LAITIGASVGGASLITGIVAA
306	tr|D7MIF7|D7MIF7_ARALL	100.0%	28.6%	FAIIIGLLAGVVLLIIFLLFL
307	tr|R0F5T3|R0F5T3_9BRAS	100.0%	28.6%	FAIIIGLLAAVVLLIIFLLFL
308	tr|M4EH70|M4EH70_BRARP	100.0%	28.6%	FAIIIGLLAAVVLLIIFLLFL
309	tr|A0A0D3BZH2|A0A0D3BZH2_BRAOL	100.0%	28.6%	FAIIIGLLAAVVLLIIFLLFL
310	tr|V4NLK4|V4NLK4_EUTSA	100.0%	28.6%	FAIIIGLLAAVVLLIIFLLFL
311	tr|A0A2C9WBX5|A0A2C9WBX5_MANES	100.0%	19.0%	FAIIIGLLAGVALIIIFLTFI
312	tr|B9SYE1|B9SYE1_RICCO	100.0%	19.0%	FAIIIGLLAGVALIIIFLTFI
313	tr|A0A2K2ART4|A0A2K2ART4_POPTR	100.0%	23.8%	FAIIIGLLAGVALLIIFLSFL
314	tr|A0A067K0M3|A0A067K0M3_JATCU	100.0%	28.6%	FAIIIGLLAGVALVIIFLMFL
315	tr|A0A2C9WK25|A0A2C9WK25_MANES	100.0%	19.0%	FAIIVGLLAGVALIIIFVNFI
316	tr|A0A2I4EC94|A0A2I4EC94_JUGRE	100.0%	23.8%	FAIIVGLLAGVALLIIFLTFV
317	tr|A0A2P6RG67|A0A2P6RG67_ROSCH	100.0%	19.0%	FAIIIGLLAGVALLIIFLTFV
318	tr|A0A251PV22|A0A251PV22_PRUPE	100.0%	23.8%	FAVIIGLLAGVALLIVFLTFI
319	tr|W9RS95|W9RS95_9ROSA	100.0%	23.8%	FAIIIGLLAGVALIIIFLAFL
320	tr|F6HSG7|F6HSG7_VITVI	100.0%	23.8%	FAIIVGLLAGVALIIIFLTFM
321	tr|A0A2R6RDZ4|A0A2R6RDZ4_ACTCH	100.0%	19.0%	FAIIIGLLAGVALLIIFLTFI
322	tr|A0A067EIS3|A0A067EIS3_CITSI	100.0%	23.8%	FAIIVGLLAGVALIIIFLTFI
323	tr|V4S8K1|V4S8K1_9ROSI	100.0%	23.8%	FAIIVGLLAGVALIIIFLTFI
324	tr|A0A0A0KQB1|A0A0A0KQB1_CUCSA	100.0%	23.8%	FAIIIGLLAGVALVIIFLVFI
325	tr|A0A0D2QHG7|A0A0D2QHG7_GOSRA	100.0%	23.8%	FAIIIGLLAGIALLIIFLAFL
326	tr|A0A1U8P435|A0A1U8P435_GOSHI	100.0%	23.8%	FAIIIGLLAGIALLIIFLAFL
327	tr|A0A1U8MJ41|A0A1U8MJ41_GOSHI	100.0%	23.8%	FAIIIGLLAGIALLIIFLAFL
328	tr|A0A1R3JCB0|A0A1R3JCB0_COCAP	100.0%	19.0%	FAIIIGLLAGIALLIIFFAFL
329	tr|A0A059AFI9|A0A059AFI9_EUCGR	100.0%	19.0%	FAIIVGLLAGVALLIIFIAFV
330	tr|A0A1Q3AXW5|A0A1Q3AXW5_CEPFO	100.0%	23.8%	FAIVIGLLAGVALLIIFLTFL
331	tr|A0A1S3UDF7|A0A1S3UDF7_VIGRR	100.0%	23.8%	FAIIIGSLAGLAILIIFLAFM
332	tr|A0A1S3UD79|A0A1S3UD79_VIGRR	100.0%	23.8%	FAIIIGSLAGLAILIIFLAFM
333	tr|A0A0L9UWC5|A0A0L9UWC5_PHAAN	100.0%	23.8%	FAIIIGSLAGLAILIIFLAFM
334	tr|V7B7W3|V7B7W3_PHAVU	100.0%	19.0%	FAIIIGSLAGAAILIIFLAFM
335	tr|I1JHV7|I1JHV7_SOYBN	100.0%	9.5%	FSIIIGSLAGVAILIIFFAFM
336	tr|I1MBU9|I1MBU9_SOYBN	100.0%	19.0%	FSIVIGSLAGVAIIIIFLAFL
337	tr|A0A1S2XKT8|A0A1S2XKT8_CICAR	100.0%	19.0%	FAIIIGLLAGVAILVILLAFL
338	tr|G7KG39|G7KG39_MEDTR	100.0%	19.0%	FALIIGLLAGAAILVILIAFL
339	tr|A0A1S3TNP5|A0A1S3TNP5_VIGRR	100.0%	23.8%	FAIIVGLLAGVAILVIFLAFL
340	tr|A0A0L9U9F1|A0A0L9U9F1_PHAAN	100.0%	28.6%	FAIIVGLLAGVAILIIFLAFL
341	tr|V7CZ31|V7CZ31_PHAVU	100.0%	28.6%	FAIIVGLLAGVAILIIFLAFL

342	tr|K7MPT1|K7MPT1_SOYBN	100.0%	28.6%	FAIIVGLLAGVAILIIFLAFL
343	tr|I1MZF0|I1MZF0_SOYBN	100.0%	28.6%	FAIIVGLLAGVAILIIFLAFL
344	tr|A0A1S2YD81|A0A1S2YD81_CICAR	100.0%	23.8%	FAIIIGLLAGVAILIIFLAFL
345	tr|G7J9C6|G7J9C6_MEDTR	100.0%	23.8%	FAIIIGLLAAVAILIIFLAFL
346	tr|A0A1U8B224|A0A1U8B224_NELNU	100.0%	19.0%	LAIIIGLLAGVALIIIFASLL
347	tr|A0A1U8ATW0|A0A1U8ATW0_NELNU	100.0%	23.8%	LAIIIGLLAGVALIIVFASFL
348	tr|A0A2G5C5U7|A0A2G5C5U7_AQUCA	100.0%	23.8%	FAITIGLLAGVALIIVFLSFI
349	tr|A0A2G5CZ38|A0A2G5CZ38_AQUCA	100.0%	19.0%	FAIIIGLLAGVALLIVFFSFV
350	tr|A0A1S3ZZV6|A0A1S3ZZV6_TOBAC	100.0%	23.8%	FALIIGLLAGVALLIIFLTFM
351	tr|A0A1U7VG50|A0A1U7VG50_NICSY	100.0%	23.8%	FALIIGLLAGVALLIIFLTFM
352	tr|A0A1J6L1U5|A0A1J6L1U5_NICAT	100.0%	23.8%	FALIIGLLAGVALLIIFLTFM
353	tr|A0A1S4AI77|A0A1S4AI77_TOBAC	100.0%	23.8%	FALIIGLLAGVALLIIFLTFM
354	tr|K4D5Q7|K4D5Q7_SOLLC	100.0%	23.8%	FALIIGLLAGVAILIIFLTFM
355	tr|M1B9Y2|M1B9Y2_SOLTU	100.0%	23.8%	FALIIGLLAGVAILIIFLTFM
356	tr|M1B612|M1B612_SOLTU	100.0%	28.6%	FALIIGLLAGVALLIIFLTFL
357	tr|K4C1F8|K4C1F8_SOLLC	100.0%	28.6%	FALIIGLLAGVALLIIFLTFL
358	tr|A0A1S3XPH2|A0A1S3XPH2_TOBAC	100.0%	23.8%	FALIIGLLAGVALLIIFLTFI
359	tr|A0A1U7WWE9|A0A1U7WWE9_NICSY	100.0%	23.8%	FALIIGLLAGVALLIIFLTFI
360	tr|A0A1J6KX62|A0A1J6KX62_NICAT	100.0%	23.8%	FALIIGLLAGVALLIIFLTFI
361	tr|A0A1S4B8L4|A0A1S4B8L4_TOBAC	100.0%	23.8%	FALIIGLLAGVALLIIFLTFI
362	tr|A0A2J6KSX7|A0A2J6KSX7_LACSA	100.0%	19.0%	FAIIIGLLAGVALIIIFLTFM
363	tr|A0A251TW13|A0A251TW13_HELAN	100.0%	23.8%	FAIIVGLLAGVALIIIFLTFM
364	tr|A0A2U1M581|A0A2U1M581_ARTAN	100.0%	23.8%	FAIIVGLLAGVALIIIFLTFM
365	tr|A0A022QWH8|A0A022QWH8_ERYGU	100.0%	19.0%	FAIIIGLLAGVALLIIFLTFV
366	tr|A0A161WVY2|A0A161WVY2_DAUCA	100.0%	28.6%	FAIIIGLLAGVALVIIFLTFL
367	tr|A0A165XSG3|A0A165XSG3_DAUCA	100.0%	19.0%	FAIIIGILAGVVLLVIFLTFL
368	tr|A0A0K9QKR4|A0A0K9QKR4_SPIOL	100.0%	33.3%	FALVIGLLAAIVIVIIFLTFL
369	tr|A0A3B6TH23|A0A3B6TH23_WHEAT	100.0%	23.8%	LAIIIGIVAAAAIIIIFLSFV
370	tr|A0A3B6SGA7|A0A3B6SGA7_WHEAT	100.0%	23.8%	LAIIIGIVAAAAIIIIFLSFV
371	tr|A0A3B6RGD4|A0A3B6RGD4_WHEAT	100.0%	23.8%	LAIIIGIVAAAAIIIIFLSFI
372	tr|A0A287E627|A0A287E627_HORVV	100.0%	23.8%	LAIIIGIVAAAAIIIIFLSFV
373	tr|C5Z7Z1|C5Z7Z1_SORBI	100.0%	38.1%	LAIIIGLVAAVAIVIVFLSFL
374	tr|A0A1D6LWS4|A0A1D6LWS4_MAIZE	100.0%	38.1%	LAIIIGLVAAAAIVIVFLSFL
375	tr|C0PA90|C0PA90_MAIZE	100.0%	38.1%	LAIIIGLVAAVAIVIVFLSFL
376	tr|A0A2T7E1A6|A0A2T7E1A6_9POAL	100.0%	33.3%	LAIIIGLVAAVAIVIVLLSFI
377	tr|K3XY89|K3XY89_SETIT	100.0%	33.3%	LAIIIGLVAAVAIVIVLLSFV
378	tr|A0A0P0WV78|A0A0P0WV78_ORYSJ	100.0%	33.3%	LAIIIGLVAAVALVIVFLSFV
379	tr|A2YBC3|A2YBC3_ORYSI	100.0%	33.3%	LAIIIGLVAAVALVIVFLSFV
380	tr|A0A0E0HNK2|A0A0E0HNK2_ORYNI	100.0%	33.3%	LAIIIGLVAAVALVIVFLSFV
381	tr|A0A0E0PVX1|A0A0E0PVX1_ORYRU	100.0%	33.3%	LAIIIGLVAAVALVIVFLSFV
382	tr|A0A0E0A7R0|A0A0E0A7R0_9ORYZ	100.0%	33.3%	LAIIIGLVAAVALVIVFLSFV
383	tr|A0A0E0LA23|A0A0E0LA23_ORYPU	100.0%	33.3%	LAIIIGLVAAVALVIVFLSFV
384	tr|A0A0D9WNX7|A0A0D9WNX7_9ORYZ	100.0%	33.3%	LAIIIGLVAAVALVIVFLSFV
385	tr|I1GZ92|I1GZ92_BRADI	100.0%	33.3%	IAIIIGLVAAVAILIIFLSFL
386	tr|M0SCL2|M0SCL2_MUSAM	100.0%	28.6%	LAIIIGLMAGVALIIVFLSFL
387	tr|M0S963|M0S963_MUSAM	100.0%	28.6%	LAIIIGLIAGVALIIVLLSFL
388	tr|M0TSV2|M0TSV2_MUSAM	100.0%	19.0%	VAIIIGLIVGVALIIFLAAFL
389	tr|A0A2H3WZ77|A0A2H3WZ77_PHODC	100.0%	19.0%	LAIIIGILAAVALILVFLSFI
390	tr|D7LRT7|D7LRT7_ARALL	100.0%	28.6%	IAIIVGVIAGFAILVVLLSLC
391	tr|V4NDE5|V4NDE5_EUTSA	100.0%	28.6%	IAIIVGVIAGFAILVVLLSLC
392	tr|A0A078IEC0|A0A078IEC0_BRANA	100.0%	23.8%	IAIIVGVIAGFAILVILLSLC
393	tr|M4DDA7|M4DDA7_BRARP	100.0%	23.8%	IAIIVGVIAGFAILVILLSLC
394	tr|A0A0D3BWL4|A0A0D3BWL4_BRAOL	100.0%	23.8%	IAIIVGVIAGFAILVILLSLC
395	tr|A0A078HTX9|A0A078HTX9_BRANA	100.0%	23.8%	IAIIVGVIAGFAILVILLSLC
396	tr|M4CTK9|M4CTK9_BRARP	100.0%	23.8%	IAIIVGVIAGFAILVILLSLC
397	tr|A0A0D3DUP0|A0A0D3DUP0_BRAOL	100.0%	23.8%	IAIIVGVIAGFAILVILLSLC
398	tr|A0A078FC27|A0A078FC27_BRANA	100.0%	23.8%	IAIIVGVIAGFAILVILLSLC
399	tr|A0A061DU08|A0A061DU08_THECC	100.0%	33.3%	VAIIVGVLAGLAVLIVLLSFC
400	tr|A0A061DU71|A0A061DU71_THECC	100.0%	33.3%	VAIIVGVLAGLAVLIVLLSFC
401	tr|A0A0D2QP86|A0A0D2QP86_GOSRA	100.0%	33.3%	VAIIVGVLAGVVVLIVLLSVC
402	tr|A0A1U8JXG4|A0A1U8JXG4_GOSHI	100.0%	33.3%	VAIIVGVLAGVVVLIVLLSVC
403	tr|A0A1U8K441|A0A1U8K441_GOSHI	100.0%	33.3%	VAIIVGVLAGVVVLIVLLSVC
404	tr|A0A2P5BEQ9|A0A2P5BEQ9_PARAD	100.0%	28.6%	VAIIVGVLAGIAVLIVLLSIC
405	tr|A0A2P5CLH2|A0A2P5CLH2_TREOI	100.0%	28.6%	VAIIVGVLAGIAVLIVLLSIC
406	tr|W9R8R9|W9R8R9_9ROSA	100.0%	28.6%	VAIIVGVLAGIAVLIVLLSIC
407	tr|A0A2H5NPS4|A0A2H5NPS4_CITUN	100.0%	38.1%	VAIIVGVVAGLAILIVFLSIC
408	tr|A0A067H1P4|A0A067H1P4_CITSI	100.0%	38.1%	VAIIVGVVAGLAILIVFLSIC
409	tr|A0A067H4B7|A0A067H4B7_CITSI	100.0%	38.1%	VAIIVGVVAGLAILIVFLSIC
410	tr|A0A1Q3CEU1|A0A1Q3CEU1_CEPFO	100.0%	33.3%	VAIIVGALAGLAVFIVLLSFC
411	tr|A0A2C9WPC7|A0A2C9WPC7_MANES	100.0%	33.3%	VAIIVGVLAGLAVLIVLLSIC
412	tr|A0A2C9VTF3|A0A2C9VTF3_MANES	100.0%	38.1%	VAIIVGIIAGLAVLIVLLSVC

413	tr|B9RD87|B9RD87_RICCO	100.0%	38.1%	VAIIVGVVAGLAVLIVLLSFC
414	tr|A0A067JXV4|A0A067JXV4_JATCU	100.0%	33.3%	VAIIVGILAGLAVLVVLLSVC
415	tr|A0A2K1XRG1|A0A2K1XRG1_POPTR	100.0%	33.3%	VAIIVGVLAGLSILIVFLSFC
416	tr|A0A2K2BIV1|A0A2K2BIV1_POPTR	100.0%	28.6%	VAIVVGILAGVAILIVLLSFC
417	tr|A0A2I4EM16|A0A2I4EM16_JUGRE	100.0%	28.6%	VAIIVGILSGVALFIVLLSIC
418	tr|A0A2I4H9Z6|A0A2I4H9Z6_JUGRE	100.0%	28.6%	VAIIVGVLAGVAVLIVLLSIC
419	tr|A0A251QGV3|A0A251QGV3_PRUPE	100.0%	42.9%	VAIIVGTVAGLAILIVILSVC
420	tr|A0A2P6SFU6|A0A2P6SFU6_ROSCH	100.0%	42.9%	VAIIVGVVASLAILIVLLSVC
421	tr|F6I389|F6I389_VITVI	100.0%	38.1%	VAIIVGVLAGLAIVVVLLSFL
422	tr|A0A1S3TFJ2|A0A1S3TFJ2_VIGRR	100.0%	28.6%	VAIIVGVFGGLAVLVVLLSIC
423	tr|A0A1S3TFJ3|A0A1S3TFJ3_VIGRR	100.0%	28.6%	VAIIVGVFGGLAVLVVLLSIC
424	tr|A0A0L9U611|A0A0L9U611_PHAAN	100.0%	28.6%	VAIIVGVFGGLAVLVVLLSIC
425	tr|V7B8Y6|V7B8Y6_PHAVU	100.0%	28.6%	VAIIVGVFGGLAVLVVLLSIC
426	tr|A0A151R5S3|A0A151R5S3_CAJCA	100.0%	28.6%	VAIIVGVFAGLAVLVVLLSIC
427	tr|K7K2J9|K7K2J9_SOYBN	100.0%	28.6%	VAIIVGVFAGLAVLVVLLSIC
428	tr|A0A1S2XQC6|A0A1S2XQC6_CICAR	100.0%	28.6%	VAIIVGVFGGLAVLVVLLSIC
429	tr|A0A1S3CC77|A0A1S3CC77_CUCME	100.0%	33.3%	VAIIIGVVAALAILVVLLSVC
430	tr|A0A0A0KEQ2|A0A0A0KEQ2_CUCSA	100.0%	33.3%	VAIIIGVVAALAILVVLLSVC
431	tr|A0A166FF49|A0A166FF49_DAUCA	100.0%	38.1%	VAIIVGVVAGIAVVIVLLSFC
432	tr|A0A1U8IVF6|A0A1U8IVF6_GOSHI	100.0%	38.1%	VALIVGVLAGLAVLIVILSFC
433	tr|A0A1U8AN72|A0A1U8AN72_NELNU	100.0%	33.3%	VAIIVGVLAALALLIVLLSFM
434	tr|A0A1U8ABG9|A0A1U8ABG9_NELNU	100.0%	33.3%	VAIIVGVLAGFALVVVLLSFL
435	tr|A0A2G5CWU3|A0A2G5CWU3_AQUCA	100.0%	28.6%	VAIIVGVLAGFAVIVVMLSLL
436	tr|A0A0L9VRT2|A0A0L9VRT2_PHAAN	100.0%	28.6%	VAIIVGVLAGLAVIVVLLSIC
437	tr|A0A1S3VD28|A0A1S3VD28_VIGRR	100.0%	28.6%	VAIIVGVLAGLAIIVVLLSIC
438	tr|V7AMU9|V7AMU9_PHAVU	100.0%	28.6%	VAIIVGVLAGLAILVVLLSIF
439	tr|A0A0R0LAI4|A0A0R0LAI4_SOYBN	100.0%	28.6%	VAIIVGILAGLAILVVILSIC
440	tr|A0A151RVB1|A0A151RVB1_CAJCA	100.0%	28.6%	VAIIVGVLAGLAILVVLLSIC
441	tr|K7KCJ2|K7KCJ2_SOYBN	100.0%	28.6%	VAIIVGVLAGLAILVVLLNLQ
442	tr|A0A0K9RL22|A0A0K9RL22_SPIOL	100.0%	38.1%	IAIIVGIIGGVAVLIVLLSVC
443	tr|A0A1S4DBQ0|A0A1S4DBQ0_TOBAC	100.0%	28.6%	VAIIVGVLAGVAVFIVLLSFC
444	tr|A0A1J6KH32|A0A1J6KH32_NICAT	100.0%	28.6%	VAIIVGVLAGVAVFIVLLSFC
445	tr|A0A1S4DFA8|A0A1S4DFA8_TOBAC	100.0%	28.6%	VAIIVGVLAGVAVFIVLLSFC
446	tr|A0A1U7W8J7|A0A1U7W8J7_NICSY	100.0%	28.6%	VAIIVGVLAGVAVFIVLLSFC
447	tr|K4AZ44|K4AZ44_SOLLC	100.0%	33.3%	VAIIVGVLAGVAVFIVLLSVC
448	tr|A0A103XUL3|A0A103XUL3_CYNCS	100.0%	33.3%	IAIVIGVVAGLTAFIVLISFC
449	tr|A0A2J6LQC9|A0A2J6LQC9_LACSA	100.0%	33.3%	IAIIIGVVAGVVAFIVLLSFC
450	tr|A0A2U1MGM4|A0A2U1MGM4_ARTAN	100.0%	33.3%	IAIIVGVVGGVAAFIILLSLC
451	tr|A0A3B6QHV7|A0A3B6QHV7_WHEAT	100.0%	23.8%	VAIIIGILAGLALLVVFISFL
452	tr|A0A3B6QIL3|A0A3B6QIL3_WHEAT	100.0%	23.8%	VAIIIGILAGLALLVVFISFL
453	tr|A0A3B6QIL6|A0A3B6QIL6_WHEAT	100.0%	23.8%	VAIIIGILAGLALLVVFISFL
454	tr|A0A3B6PQ91|A0A3B6PQ91_WHEAT	100.0%	23.8%	VAIIIGILAGLALLVVFISFL
455	tr|A0A3B6NSI1|A0A3B6NSI1_WHEAT	100.0%	23.8%	VAIIIGILAGLALLVVFISFL
456	tr|A0A287UDA2|A0A287UDA2_HORVV	100.0%	23.8%	VAIIIGILAGLALLVVFISFL
457	tr|A0A0E0GAD4|A0A0E0GAD4_ORYNI	100.0%	23.8%	VAIIIGILAGLALLVVFISFL
458	tr|A2X7Q8|A2X7Q8_ORYSI	100.0%	23.8%	VAIIIGILAGLALLVVFISFL
459	tr|I1P2J8|I1P2J8_ORYGL	100.0%	23.8%	VAIIIGILAGLALLVVFISFL
460	tr|A0A0D9YVL8|A0A0D9YVL8_9ORYZ	100.0%	23.8%	VAIIIGILAGLALLVVFISFL
461	tr|A0A0E0NIE2|A0A0E0NIE2_ORYRU	100.0%	23.8%	VAIIIGILAGLALLVVFISFL
462	tr|A0A0E0CP23|A0A0E0CP23_9ORYZ	100.0%	23.8%	VAIIIGILAGLALLVVFISFL
463	tr|A0A0E0K2Y5|A0A0E0K2Y5_ORYPU	100.0%	23.8%	VAIIIGILAGLALLVVFISFL
464	tr|K3YUJ2|K3YUJ2_SETIT	100.0%	23.8%	LAIIIGIMAGLALLVVFISFL
465	tr|A0A2T7F9P7|A0A2T7F9P7_9POAL	100.0%	23.8%	VAIIIGIMAGLALLVVFISFL
466	tr|C5XS42|C5XS42_SORBI	100.0%	23.8%	VAIAIGISAGVAALVVFISFL
467	tr|A0A1D6Q635|A0A1D6Q635_MAIZE	100.0%	23.8%	LAIAIGISAGVAALVVFISFL
468	tr|A0A0D9ZNL0|A0A0D9ZNL0_9ORYZ	100.0%	28.6%	VAIIVGILAGLAILVVFISFL
469	tr|I1PNE7|I1PNE7_ORYGL	100.0%	28.6%	VAIIVGILAGLAILVVFISFL
470	tr|Q0JBE6|Q0JBE6_ORYSJ	100.0%	28.6%	VAIIVGILAGLAILVVFISFL
471	tr|A0A0D3FYE3|A0A0D3FYE3_9ORYZ	100.0%	28.6%	VAIIVGILAGLAILVVFISFL
472	tr|A0A0E0PC29|A0A0E0PC29_ORYRU	100.0%	28.6%	VAIIVGILAGLAILVVFISFL
473	tr|A0A0E0H3M0|A0A0E0H3M0_ORYNI	90.5%	19.0%	VAIIVGILAGLAILVA--SFV
474	tr|A0A0E0KTA0|A0A0E0KTA0_ORYPU	100.0%	28.6%	VAIIVGILAGLAILVVFISFL
475	tr|A0A0E0D6L7|A0A0E0D6L7_9ORYZ	100.0%	28.6%	VAIIVGILAGLAILVVFISFL
476	tr|A0A0E0D6L8|A0A0E0D6L8_9ORYZ	100.0%	28.6%	VAIIVGILAGLAILVVFISFL
477	tr|J3LZY3|J3LZY3_ORYBR	100.0%	28.6%	VAIIVGILAGLAIIVVFISFL
478	tr|A0A1D6E4M5|A0A1D6E4M5_MAIZE	100.0%	28.6%	VAIIVGILAGLAIFVVFISFL
479	tr|C5YCY9|C5YCY9_SORBI	100.0%	28.6%	VAIIVGILAGLALFVVFISFL
480	tr|A0A2T7CXY1|A0A2T7CXY1_9POAL	100.0%	28.6%	LAIVVGILAGLALFVVFVSFL
481	tr|A0A3B6CA92|A0A3B6CA92_WHEAT	100.0%	23.8%	LAIIIGILAGLALIVVFISFL
482	tr|A0A3B6CCP3|A0A3B6CCP3_WHEAT	100.0%	23.8%	LAIIIGILAGLALIVVFISFL
483	tr|A0A3B6CAD7|A0A3B6CAD7_WHEAT	100.0%	23.8%	LAIIIGILAGLALIVVFISFL

```
484 tr|A0A3B6B1T4|A0A3B6B1T4_WHEAT 100.0%  23.8%     LAIIIGILAGLALIVVFISFL
485 tr|A0A0Q3KUA2|A0A0Q3KUA2_BRADI 100.0%  28.6%     VAILVGILAGLALIVVFISFL
486 tr|M0SRS7|M0SRS7_MUSAM         100.0%  28.6%     VAIIVGILAGLALIVVFISFL
487 tr|M0S374|M0S374_MUSAM         100.0%  33.3%     VAIIVGILAGLALIVVFLSFL
488 tr|M0RWX8|M0RWX8_MUSAM         100.0%  38.1%     VAIIVGILAGLALVVVFLSFL
489 tr|A0A2H3YCP1|A0A2H3YCP1_PHODC 100.0%  33.3%     IAIIVGIFAGVVLFVVFLSFL
490 tr|A0A2H3ZDX9|A0A2H3ZDX9_PHODC 100.0%  28.6%     VAIIVGILAGVALFVVFLSFL
491 tr|W1NFA4|W1NFA4_AMBTC         100.0%  28.6%     LAIIIGLLAGVALIIVFLSFL
```

TMD Sequence retrieval and alignment analysis method

To retrieve PDLP family, we queried UniProtKB (Release 2019_03) for members with PDLP hallmarks, two copies of the Stress-antifungal domain, excluding fragments, a TMD in the C-terminal region and also checked for signal peptide (reviewing predictions from UniProtKB, SignalP-5.0 [1] and Phobius (http://phobius.sbc.su.se/) and GPI predictions (reviewing predictions from predGPI [2] and Mendel [3]). UniRef100 [4] mapping was used to remove redundant sequences from the same organism, and NCBI BLASTp service at NCBI (BLASTP 2.9.0+ against UniProtKB/Swiss-Prot non-redundant database) was used to retrieve the best-reviewed entry hit from UniProtKB (to keep those that were PDLPs). Sequences were aligned using ClustalW included in Mega7 [5], and the alignment was edited to select the TMD. The aligned TMD was uploaded in MView tool [6] to color alignment by identity and display consensus.

PDLP-like sequences (with signal peptide, 2 Stress-antifungal domains and a TM) were retrieved by searching UniProtKB [PMID:30395287] (Release 2019_02) based on a search for entries with stress-antifungal domain, a transmembrane region prediction and lacking a kinase domain. The following query was used: database:(type:pfam PF01657) fragment:no annotation:(type:positional transmembrane) NOT (database:(type:pfam PF00069) OR database:(type:pfam PF07714)). This search retrieved a total of 533 proteins, of which 506 with two copies of the Stress-antifungal domain (*A. thaliana* PDLP1-3 and PDLP5-8 were in this set as expected); sixteen sequences contained a single copy of the domain (PDLP4 was found here), then there were some fragments (six sequences) and singletons with other domain architectures (only four sequences), but always including the Stress-antifungal domain.

The output was further refined and filtered for entries with only PF01657 (2 copies), checking for the presence of signal peptide and transmembrane via Phobius [PMID:http://phobius.sbc.su.se/] and SignalP-5.0 [PMID: 30778233], selecting those with TM in the C-terminal region, and with best BLAST hit being one of the Arabidopsis PDLP proteins (NCBI BLASTP 2.9.0+ [PMID:16218944] against UniProtKB/Swiss-Prot). Redundant sequences from the same species were removed using mapping to UniRef100 [PMID:25398609]. Finally, those with the UniProt Keyword Reference proteome were selected for subsequent sequence analysis. The collected dataset is provided in Supplementary Data 4.

The analysis that followed focused on the representative proteome set (those sequences containing the UniProtKB KW Reference proteome).

ClustalW included in Mega7 [PMID:27004904] was used to align the sequences and build phylogenetic tree. Within Mega7 software, the region containing the aligned TM membrane was selected, and saved as FASTA format to be visualized in MView. Phylogenetic analysis (Neighbor-joining tree within Mega7) and BLAST search (in NCBI against UniProtKB/Swiss-Prot) were used to guide collecting each PDLP class homolog set.

Arabidopsis thaliana PDLP1-8 sequences were retrieved from UniProtKB [PMID:30395287] (Release 2019_02). These were aligned using ClustalW included in Mega7 [PMID:27004904] and trimmed to keep the C-termini (equivalent to region 264-303 in PDLP1, Q8GXV7). This alignment was used as a query in HMMER search [PMID:29905871] to retrieve PDLP homologs. Only those with domain architecture corresponding to PDLPs were kept to compare retrieval of PDLPs.

References

1. Almagro Armenteros JJ*, et al.* SignalP 5.0 improves signal peptide predictions using deep neural networks. *Nat Biotechnol* **37**, 420-423 (2019).
2. Pierleoni A, Martelli PL, Casadio R. PredGPI: a GPI-anchor predictor. *BMC Bioinformatics* **9**, 392 (2008).
3. Eisenhaber B, Bork P, Eisenhaber F. Post-translational GPI lipid anchor modification of proteins in kingdoms of life: analysis of protein sequence data from complete genomes. *Protein Eng* **14**, 17-25 (2001).
4. Suzek BE, Wang Y, Huang H, McGarvey PB, Wu CH, UniProt C. UniRef clusters: a comprehensive and scalable alternative for improving sequence similarity searches. *Bioinformatics* **31**, 926-932 (2015).
5. Kumar S, Stecher G, Tamura K. MEGA7: Molecular Evolutionary Genetics Analysis Version 7.0 for Bigger Datasets. *Mol Biol Evol* **33**, 1870-1874 (2016).
6. Brown NP, Leroy C, Sander C. MView: a web-compatible database search or multiple alignment viewer. *Bioinformatics* **14**, 380-381 (1998).
