# Supplementary material for: An evolutionarily conserved motif is required for Plasmodesmata-located protein 5 to regulate cell-to-cell movement
Source: Commun Biol. 2020 Jun 5;3:291. doi: 10.1038/s42003-020-1007-0 (PMC7275062; doi:10.1038/s42003-020-1007-0)
Supplement: Supplementary file 2 — Description of Additional Supplementary Files [file 42003_2020_1007_MOESM2_ESM.pdf]

Description of Additional Supplementary Files

Supplementary Data 1. PDLP reference set retrieved from UniProtKB.

Supplementary Data 2. TMD dimerization prediction results using TMDOCK web server (https://membranome.org/tmdock).

Supplementary Data 3. List and sequence information for DNA primers used in this study for cloning.

Supplementary Data 4. Source data associated with graphs presented in figures 1e, 1g, 1h, 2e, 2h, 3a, 3c, 3d, 5b, and 5d, and Supplementary Figure 1b.
