# Supplementary material for: An evolutionarily conserved motif is required for Plasmodesmata-located protein 5 to regulate cell-to-cell movement
Source: Commun Biol. 2020 Jun 5;3:291. doi: 10.1038/s42003-020-1007-0 (PMC7275062; doi:10.1038/s42003-020-1007-0)
Supplement: Supplementary file 7 — Reporting Summary [file 42003_2020_1007_MOESM7_ESM.pdf]

nature research

Corresponding author(s): Jung-Youn Lee

Last updated by author(s): 4/20/2020

Reporting Summary

Nature Research wishes to improve the reproducibility of the work that we publish. This form provides structure for consistency and transparency in reporting. For further information on Nature Research policies, see Authors & Referees and the Editorial Policy Checklist.

Statistics

For all statistical analyses, confirm that the following items are present in the figure legend, table legend, main text, or Methods section.

n/a	Confirmed	
☐	☒	The exact sample size (*n*) for each experimental group/condition, given as a discrete number and unit of measurement
☐	☒	A statement on whether measurements were taken from distinct samples or whether the same sample was measured repeatedly
☐	☒	The statistical test(s) used AND whether they are one- or two-sided<br>*Only common tests should be described solely by name; describe more complex techniques in the Methods section.*
☒	☐	A description of all covariates tested
☒	☐	A description of any assumptions or corrections, such as tests of normality and adjustment for multiple comparisons
☐	☒	A full description of the statistical parameters including central tendency (e.g. means) or other basic estimates (e.g. regression coefficient) AND variation (e.g. standard deviation) or associated estimates of uncertainty (e.g. confidence intervals)
☐	☒	For null hypothesis testing, the test statistic (e.g. *F*, *t*, *r*) with confidence intervals, effect sizes, degrees of freedom and *P* value noted<br>*Give P values as exact values whenever suitable.*
☒	☐	For Bayesian analysis, information on the choice of priors and Markov chain Monte Carlo settings
☒	☐	For hierarchical and complex designs, identification of the appropriate level for tests and full reporting of outcomes
☒	☐	Estimates of effect sizes (e.g. Cohen's *d*, Pearson's *r*), indicating how they were calculated

Our web collection on statistics for biologists contains articles on many of the points above.

Software and code

Policy information about availability of computer code

Data collection	Software used for FRET analysis: Zeiss Zen 2010 D (v7.0.0.223).
Data analysis	Statistical analysis: Origin 2020 SR1 9.7.0188 (Academic).<br>Software for image processing: ImageJ bundled with 64-bit Java 1.8.0_112.

For manuscripts utilizing custom algorithms or software that are central to the research but not yet described in published literature, software must be made available to editors/reviewers. We strongly encourage code deposition in a community repository (e.g. GitHub). See the Nature Research guidelines for submitting code & software for further information.

Data

Policy information about availability of data

All manuscripts must include a data availability statement. This statement should provide the following information, where applicable:

- Accession codes, unique identifiers, or web links for publicly available datasets
- A list of figures that have associated raw data
- A description of any restrictions on data availability

All data generated or analysed during this study are included in this published article (and its supplementary information files). Proteins discussed in this work correspond to the following UniProtKB database entries: PDLP1 (Q8GXV7), PDLP2 (Q6NM73), PDLP3 (O22784), PDLP4 (Q6E263), PDLP5 (Q8GUJ2), PDLP6 (Q9ZU94), PDLP7 (Q0WPN8), PDLP8 (Q6NKQ9), BAK1 (Q94F62), ACR4 (Q9LX29).
All source data associated with graphs presented in figures 1e, 1g, 1h, 2e, 2h, 3a, 3c, 3d, 5b, and 5d, and Supplementary Figure 1b are provided in Supplementary Data 4.
All other data that support the findings of this study are available from the corresponding author upon reasonable request.

Field-specific reporting

Please select the one below that is the best fit for your research. If you are not sure, read the appropriate sections before making your selection.

☒ Life sciences ☐ Behavioural & social sciences ☐ Ecological, evolutionary & environmental sciences

For a reference copy of the document with all sections, see nature.com/documents/nr-reporting-summary-flat.pdf

Life sciences study design

All studies must disclose on these points even when the disclosure is negative.

Sample size	We selected sample size in accordance with the sample sizes used in our previous publications, data of which were validated in multiple ways.
Data exclusions	No data were excluded.
Replication	All attempts at replication was successful.
Randomization	Plant samples were randomly chosen for assays.
Blinding	Investigators were blinded to group allocation during data collection.

Reporting for specific materials, systems and methods

We require information from authors about some types of materials, experimental systems and methods used in many studies. Here, indicate whether each material, system or method listed is relevant to your study. If you are not sure if a list item applies to your research, read the appropriate section before selecting a response.

Materials & experimental systems

n/a	Involved in the study	
☒	☐	Antibodies
☒	☐	Eukaryotic cell lines
☒	☐	Palaeontology
☒	☐	Animals and other organisms
☒	☐	Human research participants
☒	☐	Clinical data

Methods

n/a	Involved in the study	
☒	☐	ChIP-seq
☒	☐	Flow cytometry
☒	☐	MRI-based neuroimaging
